# The Importance of Structural Uniformity and Chemical Homogeneity in Cobalt‐Free Lithium Excess Nickel Manganese Oxide Cathodes

**DOI:** 10.1002/advs.202300068

**Published:** 2023-04-17

**Authors:** Sven Burke, Jay F. Whitacre

**Affiliations:** ^1^ Department of Materials Science and Engineering Carnegie Mellon University Pittsburgh PA 15213 USA; ^2^ Wilton E. Scott Institute for Energy Innovation Carnegie Mellon University Pittsburgh PA 15213 USA; ^3^ Department of Engineering and Public Policy Carnegie Mellon University Pittsburgh PA 15213 USA

**Keywords:** cathode materials, cobalt‐free, energy storage, lithium‐excess, lithium‐ion batteries

## Abstract

This study explores the relationships between material quench rate during processing and the resulting structural and electrochemical properties of Li[Ni_0.25_Li_0.167_Mn_0.583_]O_2_. Samples of this lithium‐rich material are prepared with highly contrasting postfiring cooling methods: a rapid water emersion quench or closed‐door oven cooling. The contrasting approaches result in samples with different structural, chemical, and electrochemical behaviors; after cycling the rapidly quenched material yields greater capacity, greater stability, and initially lower, but more stable voltages than the slower cooled samples. Through the use of scanning tunneling electron microscopy, X‐Ray Diffraction (XRD), and X‐ray photoelectron spectroscopy (XPS) it is demonstrated that rapidly quenched powders are more structurally uniform and chemically homogenous before cycling. By comparing these precycling sample to postcycling samples, it is then examined how this increased structural uniformity and chemical homogeneity leads to the superior electrochemical properties of the rapidly quenched samples.

## Graphical abstract

Rapidly quenching Li[Ni_0.25_Li_0.167_Mn_0.583_]O_2_ cathode material in water results in a chemically homogenous and structurally uniform material when compared to slowly cooled samples. The chemical homogeneity of the rapidly cooled samples results in cathodes that yielded greater stability and higher specific capacity over many cycles. The possible mechanisms for this are also discussed.

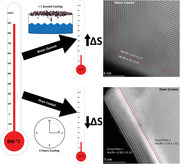

## 1 Introduction

Lithium‐containing ceramic metal oxides, such as LiMO_2_ (where M denotes a transition metal such as Nickle, Manganese, Cobalt, etc.), are sensitive to their synthesis conditions.^[^ ^2^ ^]^ Cooling rates after high temperature processing are a critically important, and understudied, part of cathode synthesis, and previous results indicate that lithium excess cathode materials are particularly sensitive to cooling rate.^[^ ^1^ ^]^ In this investigation, we explore the impacts of cooling rate on the chemical and structural homogeneity of the resultant lithium excess cathodes.^[^ ^1^, ^3^, ^4^ ^]^

Several contemporary cathodes, such as Li[Ni*_x_* Mn_(1/2−_ *_x_*_/2)_Co_(1/2−_ *_x_*_/2)_]O_2_ (NMC) where 0.33 ≤ *x* ≤ 0.8 and Li[Ni*_x_* Co*_y_* Al*_z_*]O_2_ where *x* + *y* + *z* = 1 (NCA), are derivates of the lithium cobalt oxide cathode first described by John B. Goodenough.^[^ ^5^, ^6^ ^]^ These oxides have a R‐3m layered structure where there are alternating layers of lithium and transition metals with a layer of oxygen between. In this configuration, the original LiCoO_2_ (LCO) chemistry derives electrochemical capacity from the intercalation of lithium and the associated redox of the Co^3+/4+^ ion pair.^[^ ^5^, ^7^, ^8^ ^]^ Critically in this LCO chemistry, the cobalt atoms are both the active redox species as well as the primary structurally stabilizing species. In recent years, the economic and ethical costs of cobalt have sparked a need for reducing and eventually eliminating the cobalt.^[^ ^9^, ^10^, ^11^ ^]^ The use of the Ni^2+/3+/4+^ redox series as the primary active species in cathodes allowed for a significant reduction in the amount of cobalt in cathodes, however the large variance in the ionic size of nickel ions makes these cathodes rather unstable.^[^ ^12^, ^13^, ^14^, ^15^ ^]^ Lattice bound cobalt has a highly stable crystal field which helps preserve the lattice structure in these cathodes over the course of cycling.

One solution is to use lithium excess chemistries, which have been reported in the literature since the late 1990's and have recently gained more attention. For this study, we chose the cobalt‐free Li[Ni*_x_* Li_(1/3−2_ *_x_*_/3)_Mn_(2/3−_ *_x_*_/2)_]O_2_ materials system where *x* = 0.25; as synthesized, Li[Ni_1/4_Li_1/6_Mn_7/12_]O_2_. The layered structure of alternating lithium and transition metals is upset by the presence of excess lithium in the transition metal layer.^[^ ^4^, ^16^, ^17^, ^18^, ^19^ ^]^ The low oxidation state, +1, of the excess lithium in the transition metal layer helps stabilize the manganese as Mn^4+^, and therefore helps stabilize the layer structure of the materials despite the Ni^2+/3+/4+^ redox.^[^ ^3^, ^7^, ^13^, ^15^, ^20^, ^21^ ^]^ These materials also have higher capacities than theory would suggest, due to the formation of Li—O—Li bonds between the layers which allow for the redox of the lattice oxygen; the formation of these bonds is greatly aided by the inclusion of lithium in the transition metal layer.^[^ ^22^, ^23^, ^24^, ^25^, ^26^, ^27^ ^]^ However, despite these theoretical advantages this family of cathodes has been plagued by low‐rate capabilities, and capacity and voltage fade that are related to structural instability, which many previous works have sought to address.^[^ ^12^, ^28^, ^29^, ^30^, ^31^, ^32^, ^33^, ^34^, ^35^ ^]^

Recent work has determined one of the possible root causes of these shortcomings is the underlying inhomogeneity of the material.^[^ ^36^ ^]^ This underlying inhomogeneity of lithium excess chemistries was reported in 1998 by Neudecker et al. where (to our knowledge) the first X‐Ray Diffraction (XRD) pattern of this class of cobalt‐free lithium‐rich materials was published. Most of the peaks in the pattern indexed to the R‐3m structure, and the peaks between 20 and 25 °2*θ* indexed to the C2/m structure.^[^ ^16^ ^]^ From this Neudecker proposed the structure of these materials to be a mixture of a LiNiO_2_ like R‐3m phase and a Li_2_NiO_3_ like C2/m phase. Later work on these materials would then propose that the origin of the 20 and 25 °2*θ* peaks was super lattice ordering in the transition metal layer and not separate regions of the monoclinic C2/m phase.^[^ ^4^, ^18^, ^19^, ^21^, ^37^ ^]^ Since then, the exact structure of these materials has been debated and, indeed, results that show that the material takes different crystallographic forms depending on the precursors and synthetic routes.^[^ ^3^, ^4^, ^12^, ^15^, ^16^, ^18^, ^19^, ^21^, ^22^, ^38^, ^39^, ^40^, ^41^, ^42^, ^43^, ^44^ ^]^

The literature frequently reports that the differences in the structure are, in great part, the result of differing synthetic routes, and/or variance in the utilized synthetic steps and conditions.^[^ ^1^, ^4^, ^15^, ^22^, ^41^, ^42^ ^]^ The ambiguity concerning whether these materials consist of nanocomposites of Li_2_MnO_3_ and LiNi_.5_Mn_0.5_O_2_, or aare instead a single‐phase/solid solution material, has made it difficult to accurately attribute the various shortcomings these materials exhibit to any root causes. Despite this, previous investigations have revealed several methods for postproduction processing that can mitigate or eliminate some of the challenges of low‐rate capabilities, capacity and voltage fade, and structural instability.^[^ ^12^, ^28^, ^29^, ^30^, ^31^, ^32^, ^33^, ^34^, ^35^ ^]^ Similar shortcomings in NMC also results in complex synthesis techniques, where purposely heterogeneous materials were made through complex synthesis, further demonstrating the key connections between synthetic techniques, and material homogeneity.^[^ ^45^, ^46^, ^47^ ^]^ All of these synthesis methods are inventive and insightful, but they have proven to be complex and expensive, making scalability an ever‐present question. In past investigations, we demonstrated the addition of water quenching to a common sol–gel synthesis resulted in materials with excellent capacity and cycle stability.^[^ ^1^ ^]^ Through combing our previous work on quenching Li[Ni*_x_* Li_(1/3−2_ *_x_*_/3)_Mn_(2/3−_ *_x_*_/3)_]O_2_ cathodes with a previous investigation from Dahn et al., which found slower cooling rates cause phase segregation in the LiCoO_2_–Li_2_MnO_3_ composites systems, we seek to explore the impacts of cooling rate on the Li[Ni*_x_* Li_(1/3−2_ *_x_*_/3)_Mn_(2/3−_ *_x_*_/3)_]O_2_ system.^[^ ^48^ ^]^ While the LiCoO_2_–Li_2_MnO_3_ system and Li[Ni*_x_* Li_(1/3−2_ *_x_*_/3)_Mn_(2/3−_ *_x_*_/3)_]O_2_ system undoubtedly have some similarities, the Li[Ni*_x_* Li_(1/3−2_ *_x_*_/3)_Mn_(2/3−_ *_x_*_/3)_]O_2_ system used in this investigation lacks cobalt, which as previously mentioned helps with the stabilization of the material's layered structure. This investigation also constitutes a logical extension of our previous work by exploring the structural, chemical, and electrochemical effects of two extreme cooling rates, natural oven cooling (referred to as OC for the rest of this investigation), and rapid water quenching (referred to as WQ for the rest of this investigation), and the use of these cooling rates provides a greater difference in cooling than previously explored rates. Additionally, this work seeks to correlate the effects of electrochemical cycling on the structure and chemistry of these two differently cooled materials (adding the suffix “pre” for samples before cycling, and “post” for samples after cycling).

Through examining the differences between these two materials, and how cycling impacts them, we aim to elucidate the mechanism by which the rapid water quenching of these lithium excess materials improves performance and stability, and to clearly describe our work, we offer the following definitions for interlayer ordering and intralayer ordering (**Table** **1**). Interlayer ordering: A measure of the number of Li+/Ni2+ antisite defects present in the material. Less antisite defects mean higher interlayer order and more antisite defects means lower interlayer order. This is measured through taking the ratio of the (003) and (104) peaks’ intensities, where a higher peak ratio means less antisite defects and more ordering. Intralayer ordering: The ordering in the transition metal layer (TM). More localized excess‐Li systems, using terminology from Kang et al.,^[^ ^49^ ^]^ means more intralayer ordering, and more delocalized excess‐Li systems means less intralayer ordering. Using other terminology, the formation of the hexagonal structure within the transition metal layer mentioned in previous investigations,^[^ ^4^, ^21^ ^]^ is akin to the more delocalized excess‐Li systems and therefore is more disordered. This is measured by the ratio of sum of the (006) and (102) peaks’ intensities to the intensity of the (101) peak, where the higher the ratio the more ordering in the system.^[^ ^4^, ^21^, ^50^, ^51^, ^52^, ^53^, ^54^ ^]^

**Table 1:** Terminology and abbreviations

Abbreviation or term	Definition
TM	Transition Metals, for this investigation specifically Mn and Ni.
Li_Li_	The lithium found in the lithium layers.
Li_XS_	The excess lithium found in the transition metal layer.
Li_AS_	Lithium found in the transition metal layers as a result of anti‐site defects.
Ni_AS_	Nickel found in the lithium layers as a result of antisite defects.
WQ	Water quenched, taken from a hot furnace an brough to room temperature by inverting over a water bath.
OC	Oven cooled, allowed to come to room temperature in a close door furnace.
x‐pre	Short for “precycling,” indicates that sample “x” is pristine and not yet cycled.
x‐post	Short for “postcycling,” indicates that sample “x” has undergone cycling.

For this investigation, it is important to note the relationships between these concepts and note where in this investigation they will apply. In the case of this investigation, the systems with greater entropy are the more disordered systems, for example an increase in the number of antisite defects means the system has a higher entropy and thus is less ordered. Additionally, the more entropy systems will have a greater degree of chemical homogeneity, for example more antisite defects means there is a more even distribution of Li and Ni across the layers of the material which means it is more homogenous.^[^ ^48^ ^]^ The extension of this is that the segregation of phases or elements results in a decrease in entropy and therefore is a more ordered system.

## 2 Results and Discussion

### 2.1 Before Cycling Characterization

**Figure 1:**
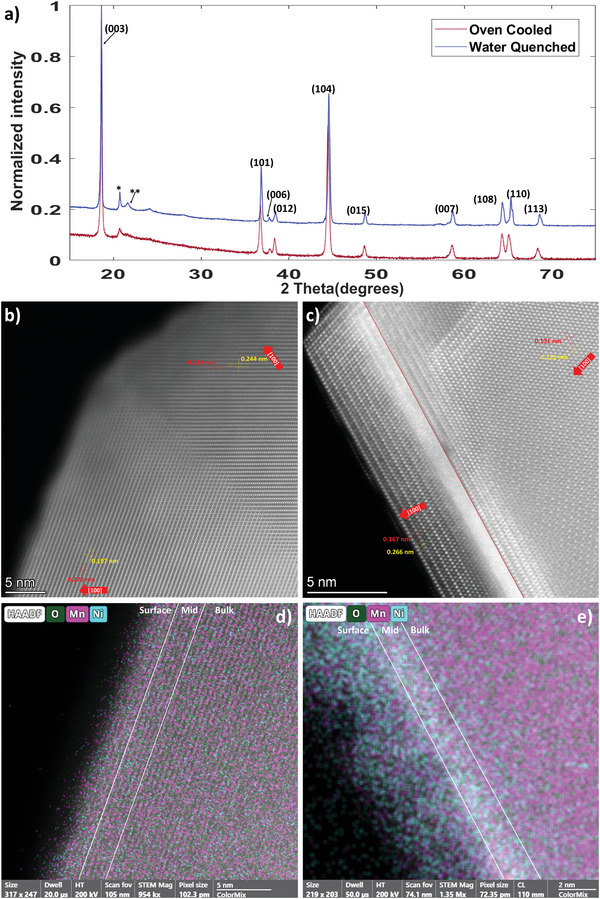
a) XRD patterns of the OC‐pre, and WQ‐pre samples with peaks indexed to the R‐3m structure, and * and ** indexing the superlattice peaks. b) STEM micrograph of the WQ‐pre sample. c) STEM micrograph of the OC‐pre sample, where two images of the sample taken at two focus points were knitted together, the red line demonstrates where the change in focus is. d) EDS map of the WQ‐pre sample with oxygen, nickel, and manganese as shown. e) EDS map of the OC‐pre sample with oxygen, nickel, and manganese as shown. Please refer to Tables 2 and 3 for more details.

The XRD patterns of the water quenched sample, before electrochemical cycling (WQ‐pre) and (oven cooled sample, before electrochemical cycling (OC‐pre) seen in **Figure** **1**a both index to the R‐3m structure with the exceptions of the superlattice peaks, marked * and **, which are indicative of these lithium excess materials. The superlattice peaks in the XRD pattern of the WQ‐pre sample are more well defined than in the XRD pattern of the OC‐pre sample, which is indicative of intralayer disorder in the WQ‐pre sample. The XRD patterns of the both samples, see the Supporting Information 2, also indicate that the WQ‐pre sample has a more disordered transition metal layer than the OC‐pre sample^[^ ^4^, ^21^, ^50^ ^]^; this suggests that the rapid water quenching of the system creates more disorder in the transition metal layer. However, Supporting Information 2 also indicates that sample WQ‐pre has less Li^+^/Ni^2+^ antisite defects than the OC‐pre sample,^[^ ^50^ ^]^ indicating that water quenching reducing the amount of antisite defects and increase inter‐layer order. The refinement of the two XRD patterns can also be seen in the Supporting Information 19. The Chemo‐structural differences between these two samples were also apparent to the naked eye. As seen in the Supporting Information 3, the two samples visually are different colors, and the WQ‐pre sample has a higher red value than the OC‐pre sample. This visual color contrast between the two samples is potentially a helpful metric as the color of these samples is determined by chemical and structural aspects of the material.

The scanning transmission electron microscopy (STEM) micrographs seen in Figure 1b,c demonstrate that the WQ‐pre particle is a structurally uniform single‐phase particle and that the OC‐pre particle consists of a minimum of two an‐isostructural phases, a surface phase and a bulk phase, with potentially an intermediate phase/grain boundary separating them. The micrograph of the WQ‐pre particle has two different layer orientations intersecting at the vertex, as seen in the Supporting Information 5 these layered regions are aligned in orientations of the same family, indicating these layered regions are at the very least isostructural.

In comparison, the OC‐pre sample has three distinct phases, a surface phase, a bulk phase, and an intermediate phase between them. The surface phase has clear layers of alternating intensity, the bulk phase is a more uniform intensity, and the intermediate phase is the most intense. As can be seen in the left most part of bulk phase focus area in Figure 1c, the intermediate phase is isostructural with the bulk phase, but is more closely packed, though it has more clearly defined atomic layers of alternating intensity. This intermediate phase sharing characteristics of both the surface and bulk phases suggests it might be more similar to a grain boundary than a distinct third phase.

The structural difference between the structurally uniform and phase pure WQ‐pre sample and the two‐phase OC‐pre sample is likely a result of the 5 orders of magnitude difference in sample cooling rate seen in the Supporting Information 1. Since the R‐3m phase is only meta‐stable at room temperature the rapid cooling experienced by the WQ‐pre sample would stabilize the R‐3m phase. The slow cooling of the OC‐pre sample would be gradual enough to allow for the meta‐stable R‐3m to decay and being to phase segregate into two distinct phases (**Tables** **2** and **3**).

**Table 2:** This table shows the EDS results of the WQ‐pre sample seen in Figure 1f, with results categorized by the region of the image

EDS results of WQ‐pre					
Region	Mn at%	Ni at%	O at%	Mn/Ni	O/(Mn+Ni)
Surface	19.43 ± 1.94	4.03 ± 0.40	51.36 ± 5.14	4.82	2.19
Middle	26.16 ± 2.62	3.78 ± 0.38	61.56 ± 6.16	6.92	2.06
Bulk	24.58 ± 2.46	3.09 ± 0.30	65.00 ± 6.50	7.95	2.35
All	24.21 ± 2.42	3.24 ± 0.32	62.94 ± 6.29	7.47	2.29

**Table 3:** This table shows the EDS results of the OC‐pre sample seen in Figure 1e, with results categorized by the region of the image

EDS results of OC‐pre					
Region	Mn at%	Ni at%	O at%	Mn/Ni	O/(Mn+Ni)
Surface	26.36 ± 2.63	29.54 ± 2.95	44.10 ± 4.41	0.89	0.79
Middle	30.15 ± 3.02	17.16 ± 1.72	52.70 ± 5.27	1.76	1.11
Bulk	38.02 ± 3.80	4.13 ± 0.41	57.85 ± 5.79	9.21	1.37
All	33.02 ± 3.30	12.64 ± 1.26	54.33 ± 5.43	2.61	1.19

The energy dispersive spectroscopy (EDS) results seen in Figure 1d,e demonstrate that there are distinct chemical differences between WQ‐pre and OC‐pre. WQ‐pre is far more chemically homogenous than OC‐pre, with bulk of WQ‐pre being 1.65 times more manganese rich than the surface, while the bulk of OC‐pre is 10.35 times more manganese rich than the surface. These data demonstrate that the phase segregation seen in the OC‐pre sample result in a relatively nickel‐rich surface phase and a relatively manganese‐rich bulk phase, while the structural uniform WQ‐pre sample does not see significant elemental segregation within its single phase. These data are consistent with previous investigations that have reported elemental and phase segregation in similar compounds. However, these investigations omitted specifics on sample cooling, so it is assumed the samples were cooled more similar to the natural oven cooling.^[^ ^15^, ^50^, ^55^, ^56^, ^57^, ^58^ ^]^ The elemental make‐up of the two phases seen in the OC‐pre sample also support the phase decomposition of a chemically homogenous R‐3m phase into two chemically distinct LiMn_0.5_Ni_0.5_O_2_ like and Li_2_MnO_3_ like phases over the course of the gradual cooling.^[^ ^40^ ^]^ Similarly, the relative chemical homogeneity of the WQ‐pre sample further supports the stabilization of the R‐3m phase through rapid water quenching.

The relative oxygen content of WQ‐pre and OC‐pre are also quite different. The ratio of oxygen to transition metals (O/TM ratio) in the WQ‐pre sample are much more uniform and closer to the theoretical value of 2.4 than the O/TM ratio seen in the OC‐pre sample. The relative oxygen content of the whole OC‐pre sample is roughly half of the theoretical value, but the nickel rich surface of the OC‐pre sample is far much oxygen‐poor than the rest of the OC‐pre sample. The reason for this difference in relative oxygen content is unknown, though it is likely also a result of phase and elemental segregation.

The EDS and XRD characterization of the pristine materials are consistent with each other, with EDS suggesting chemical homogeneity of WQ‐pre and XRD suggesting intralayer disorder, and therefore also chemical homogeneity. Interestingly the inter‐layer disorder for the OC‐pre sample is higher than the WQ‐pre sample, which would suggest greater nickel homogeneity, which contradicts EDS observations. The phase segregation of the OC‐pre would mean any XRD of the sample would likely be dominated by the manganese‐rich bulk phase, which from EDS observation is quite internally homogenous.

### 2.2 Comparing Electrochemical Performance

**Figure 2:**
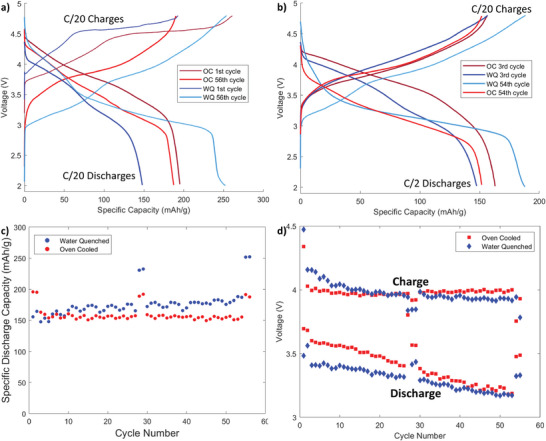
a) The first and last cycling curves from the WQ and the OC sample, both symmetric C/20 rate. b) the 3rd and 54th cycling curves from the WQ and OC samples with a C/20 charge and a C/2 discharge. c) The discharge capacity per cycle for the WQ and the OC sample, oscillation in capacity is due changes in temperature in the room. d) The capacity averaged discharge voltage for the WQ and OC sample over the course of cycling. The charge and discharge resistances as well as impendence corrected average voltage plots of these samples can be found in the Supporting Information 20.

The structural and chemical difference between WQ‐pre and OC‐pre resulted in notable differences in electrochemical performance. As seen in **Figure** **2**, the initial specific discharge capacity of OC was 40 mAh g^−1^ higher than WQ. The first charge/formation cycle of the samples resulted in proportionally similar amounts of irreversible capacity loss of 45% and 48% of initial capacity for WQ and OC, respectively. The primary difference in formation cycling was the higher voltage of the plateau observed for the WQ sample, which was on the order of 50 mV higher than the plateau of the OC sample. As demonstrated in Figure 2a,c, the specific discharge capacity of the WQ sample grew by around 100 mAh g^−1^ by the 56th cycle, while the OC sample lost 10 mAh g^−1^ of capacity. These results are consistent with our previous investigation's findings that more rapidly quenched samples demonstrated increased capacity and capacity growth.^[^ ^1^ ^]^

Similarly, the voltage fade behaviors of both samples, see Figure 2d, are also consistent with our past investigation. The WQ sample starts with an initially lower capacity‐averaged discharge voltage, but over the course of cycling, the voltage faded at a slower rate than the OC sample's, with voltage fading rates of roughly 4  and 7 mV per cycle for WQ and OC, respectively (see the Supporting Information 7). The initially higher discharge voltage of the OC sample is nearly identical to the WQ's by the end of cycling. When cycled at a rate of C/20 the voltage fade seen in both samples is mitigated, though OC still experiences a greater degree of voltage loss. These data are consistent with recent work that attributes voltage fade to the lattice strain caused by sample heterogeneity.^[^ ^36^ ^]^ However, the charge voltages of the samples are not consistent with this explanation, as the charge voltage of the OC sample is very consistent with minimal fading, while the charge voltage of the WQ sample resembles the fading seen in the WQ sample's discharge voltages.

### 2.3 After Cycling Characterization

**Figure 3:**
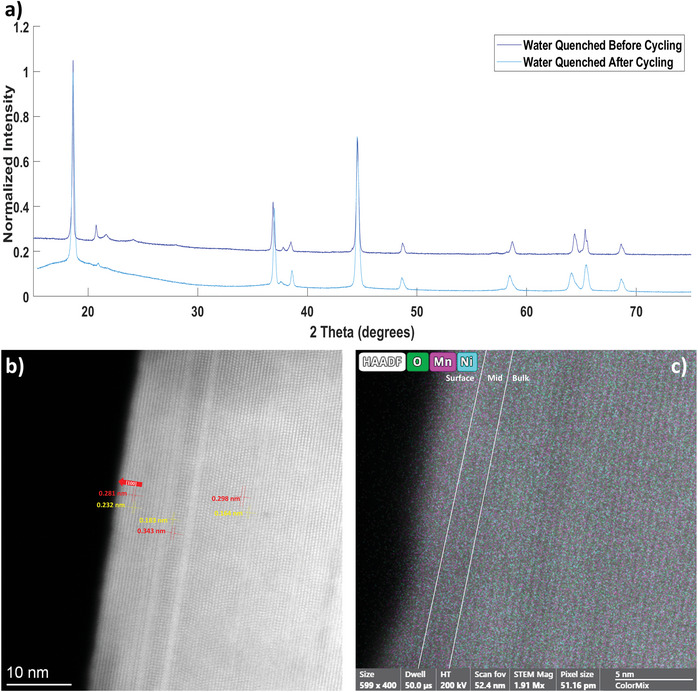
a) XRD patterns of the WQ sample before and after cycling, post‐cycling sample's low angle scatter is due to the binder and carbon black in the sample. b) STEM micrograph of the WQ‐post sample. c) EDS map of the WQ‐post sample with oxygen, nickel, and manganese as shown. Please refer to Table 4 for more details.

**Figure 4:**
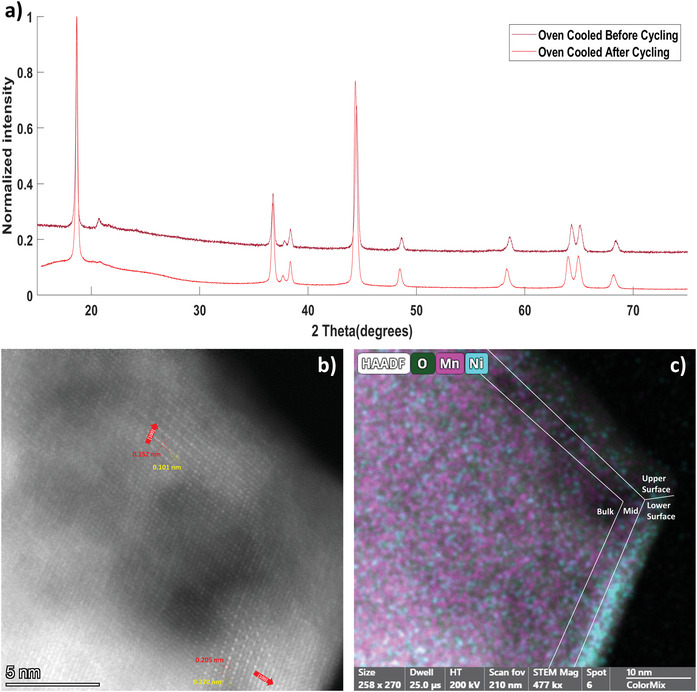
a) XRD patterns of the OC sample before and after cycling, postcycling sample's low angle scatter is due to the binder and carbon black in the sample. b) STEM micrograph of the OC‐post sample. c) EDS map of the OC‐post sample with oxygen, nickel, and manganese as shown. Please refer to Table 4 for more details.

Despite the different electrochemical performance of the WQ and OC samples the XRD patterns of both samples, see **Figures** **3**a and **4**a, suggest that they experience similar structural and chemical changes from cycling. This similarity is change is further confirmed through the refinement of the samples seen in the Supporting Information 19, where it is clear both samples experienced a significant degree of structural rearrangement as a result of cycling. Both samples see a loss of super lattice peaks, indicating movement of the transition metals over the course of cycling, which is consistent with previous investigations. The peak ratios of the XRD patterns, see the Supporting Information 8, indicate that both samples saw decreases in interlayer, with more antisite defects formed during cycling. Supporting Information 8 also indicates both samples lost intralayer order as well, which is consistent with the increased prevalence of antisite defects; having more Li_AS_ (anti‐site lithium) and Ni_AS_ (anti‐site nickel) results in more entropic, and thus disordered transition metal layers. These data demonstrate that the electrochemical migration of transition metal ions drives the sample toward greater homogeneity and therefore is entropically driven. This indicates that the greater performance and cycle life of the WQ sample is a result of the sample's homogeneity, and therefore high entropy initial state (**Table** **4**).

**Table 4:** This table shows the EDS results for the water quenched sample after cycling based off the region of the image

WQ‐Post					
Region	Mn at%	Ni at%	O at%	Mn/Ni	O/(Mn+Ni)
Surface	29.93 ± 2.99	8.78 ± 0.88	48.61 ± 4.86	3.41	1.26
Middle	26.20 ± 2.62	10.50 ± 1.05	54.21 ± 5.42	2.50	1.48
Bulk	26.75 ± 2.68	9.00 ± 0.90	55.77 ± 5.58	2.97	1.56
All	26.52 ± 2.65	9.04 ± 0.90	53.84 ± 5.38	2.93	1.51

The STEM of the WQ‐post (Water Quenched sample, after electrochemical cycling) sample, Figure 3b, has evidence of dislocations in the particle, indicating that electrochemical cycling caused some structural alteration. However, despite these features, the layered structure of the WQ‐post materials is still intact demonstrating that an increase in dislocation density does not cause structural collapse. Additionally, the high‐resolution EDS results of WQ‐post show that the dislocations are not correlated with chemical changes in the sample either. The WQ‐post sample exhibited a more uniform Mn/Ni ratio than the WQ‐pre sample. The EDS also indicates that the sample maintained a homogenous oxygen to transition meal (O/TM) ratio, where TM is used as a stand‐in for "transition metal", though this ratio is lower than observed in the WQ‐pre sample; this is expected as previous studies have demonstrated that cycling, particularly activation cycling, results in the loss of oxygen from the lattice (**Table** **5**).^[^ ^52^, ^59^ ^]^

**Table 5:** This table shows the EDS results for the oven cooled sample after cycling based off the region of the image

OC‐Post					
Region	Mn at%	Ni at%	O at%	Mn/Ni	O/(Mn+Ni)
Top surface	11.89 ± 1.19	2.20 ± 0.22	85.91 ± 8.59	5.40	6.10
Lower surface	15.61 ± 1.56	9.41 ± 0.94	74.97 ± 7.50	1.66	3.00
Middle	22.49 ± 2.25	2.37 ± 0.24	75.14 ± 7.51	9.49	3.02
Bulk	25.13 ± 2.51	2.42 ± 0.24	72.45 ± 7.25	10.38	2.63
All	23.79 ± 2.38	2.79 ± 0.28	73.42 ± 7.34	8.53	2.76

The STEM seen in Figure 4b shows that the two‐layer orientations of the OC‐post (Oven Cooled sample, after electrochemical cycling) particle are isostructural and intersect at the vertex of the particle. Additionally, as evidenced by the varying thickness of the particle, there was some degree of structural degradation observed as a result of cycling, though not enough to cause complete structural collapse. Additionally, the EDS of the sample reveals that while OC sample postcycling is more chemically homogeneous than the OC‐pre sample, it is still chemically heterogenous with the Mn/Ni ratio of the lower surface being more than 3 times that of the lower surface, and the bulk's Mn/Ni ratio roughly 2 and 6 times higher than the upper and lower surfaces, respectively. The O/TM ratio of the sample is more homogenous with all regions but the upper surface having an O/TM ratio roughly between 2.5 and 3. The upper surface, however, has a O/TM ratio of 6.10, which is significantly higher than the rest of the sample. The relatively high O/TM ratio could be the result of two things: increased oxygenation of the sample, or the loss of transition metals. The XPS of the OC sample before and after cycling demonstrates that the OC samples sees more vacancies form over the course of cycling, which suggests oxygen is lost in cycling, not gained. The XPS (X‐ray Photoelectron Spectroscopy) signal for lattice bound oxygen loses intensity as a result of cycling, further suggesting that the system does not create new O—TM bonds.

The loss of nickel through transition metal dissolution would explain the high Mn/Ni ratio seen in the upper surface of the OC‐post particle, see Figure 4. The loss nickel would be consistent with how Ni^2+^ is more prone to dissolution than Mn^4+^.^[^ ^45^, ^60^ ^]^ Dissolution of the nickel‐rich surface phase would also help explain the pitting seen in the STEM of the sample, and could also explain the lack of a distinct surface phase. The XPS of the OC sample seen in the Supporting Information 14–17 also support the theory that there is some dissolution of cathode materials, as many of the TM oxidations states present in the OC‐post sample are highly soluble. Additionally, the presence of these TM oxidation states, such as Mn^3+^, form due to charge compensation from losing the low voltage Ni^2+^, and the Mn^4+^ to Mn^3+^ transition results in Jahn–Teller distortions, which would only expedite the dissolution and structural collapse of the material. The OC‐post sample also saw the nucleation of a cubic defect phase, with the EDS data indicating that the phase is relatively high in nickel (see the Supporting Information 12). These results are consistent with the theory that there has been nucleation of a spinel phase, which is a common failure and aging mechanisms of nickel rich cathodes.^[^ ^61^, ^62^, ^63^, ^64^ ^]^

The results from Figures 3 and 4 can help explain the difference in electrochemical behavior seen in the WQ and OC samples. The relatively more rapid voltage fade of the OC sample is consistent with the nucleation of the low voltage spinel‐like defect phases. The nucleation of spinel and the dissolution of cathode material, with preferential nickel‐rich dissolution, seem to correlate with the reduced cycle stability observed in the OC materials.

The WQ sample, by contrast, likely gained capacity as a result of the migration of transition metals in the material allowing for the formation of Li—O—Li bonds over the course of cycling. This is supported by the XPS data seen in the Supporting Information 13 and by the consistent evidence of cycling causing transition metals to migrate not only in the transition metal layers, but also between the lithium and transition metal layers.

The data suggest that the WQ‐pre sample is more disordered and therefore more chemically homogenous than the OC‐pre sample, with the WQ‐pre sample having more antisite defects, greater intralayer homogeneity, and only one uniform phase. A homogenous phase‐pure material, like WQ‐pre, would have even distributions of Ni^2+^_,_ Mn^4+^_,_ and Li^+^ ions throughout the transition metal layer. This homogeneity means the Ni^2+/4+^ redox will be more evenly distributed through the material, causing less localized lattice stress.^[^ ^36^ ^]^ The homogeneity will also mean each Mn^4+^ ion will be close by to a stabilizing Li^+^ ion, which will prevent the manganese from reducing into Mn^3+^, where manganese would be both electrolyte soluble and Jahn–Teller active.^[^ ^52^, ^62^, ^65^, ^66^ ^]^

This mechanism also provides insight in the failure of the OC sample. The phase segregation of the sample into a nickel‐rich surface phase and a manganese‐rich bulk phase would prevent Mn^4+^ from reducing the lattice stress of the Ni^2+/4+^ redox, and would also make the Li^+^ stabilization of Mn^4+^ less effective. This would result in a Ni‐rich phase with a high degree of lattice strain, which would accelerate the dissolution and rapid structural degradation of the Ni‐rich phase.^[^ ^36^, ^60^ ^]^ This would also see the formation of Mn^3+^ and subsequent dissolution and degradation of active material associated with Jahn–Teller active manganese.^[^ ^52^, ^62^, ^65^, ^66^ ^]^

These results indicate that the water quenching of Li[Ni*_x_* Li_(1/3−2_ *_x_*_/3)_Mn_(2/3−_ *_x_*_/3)_]O_2_ material stabilizes the meta‐stable R‐3m phase. The data demonstrate the resultant phase is the more chemically homogenous, and therefore exists at a higher entropy than the thermodynamically cooled sample; this mirrors the definition of a metastable phase existing at a local entropy maximum and is consistent with previous results.^[^ ^48^, ^67^ ^]^ Additionally, the entropically driven degradation of the materials has a greater impact on the thermodynamically stable OC material than on the WQ material. This demonstrates that the WQ material likely exists near the entropy maximum of the system, which would account for why it requires higher energy inputs during charging and outputs greater energy on discharge. Further, this provides insight into how to reduce the degradation of these lithium excess materials, determine the point at which their chemistry exists at entropy maximum, and therefore energy minimum, and then make these materials as homogenous as possible through stabilizing this high‐entropy meta‐stable phase.

### 2.4 Explanation for the Gradual Capacity Increase of the WQ Sample

Over the course of the 56 electrochemical cycles seen in Figure 2 the WQ sample saw a gradual increase in capacity. The data of this investigation indicate that the electrochemical cycling of Li[Ni*_x_* Li_(1/3−2_ *_x_*_/3)_Mn_(2/3−_ *_x_*_/3)_]O_2_ material results in increasingly homogenous material. We propose that as a side‐effect of this effect is the gradual formation of Li—O—Li bonds. The formation of more Li—O—Li bonds would require increasing amounts of lithium moving from the lithium layer (Li_Li_) to the transition metal layer (Li_TM_). Through comparing the I_(003)_/I_(104)_ ratios of the WQ material before and after cycling, it is clear that cycling caused the formation of more antisite defects, demonstrating that the Li^+^ ions in the material are moving across both the lithium and the TM‐layers, which would result in more Li_TM_. However, the comparison of the I_(003)_/I_(104)_ ratios for the OC sample also support the formation of antisite defects from cycling, and yet this sample does not see an increase in capacity.

XPS on the samples, Supporting Information 14, reveal the presence of the oxygen state associated with Li—O—Li bonds in the WQ material, while this oxygen state is not present in the OC material. This reveals that the formation of Li—O—Li bonds is linked not only to the presence of lithium in the transition metal layer, but also possibly to the chemical homogeneity of the sample. The distinct phase segregation seen in the OC‐pre sample would likely result in a majority of the antisite defects occurring in the nickel‐rich surface phase, whereas some literature suggests antisite defects would cause rapid degradation in the OC sample preventing the formation of any beneficial Li—O—Li bonds^[^ ^68^, ^69^, ^70^ ^]^; and as previously discussed Figure 4 provides evidence of degradation in the OC sample. Additionally, there is a growing body of evidence that during electrochemical activation the surfaces Li‐excess materials experience irreversible loss of oxygen and lithium from the surfaces instead of forming Li—O—Li bonds seen in the bulk of the material.^[^ ^71^, ^72^, ^73^ ^]^ The increase in capacity seen in the cycling of the WQ therefore suggest that the sample's chemical homogeneity counteracts these forms of degradation and provides a stable bulk phase for the gradual formation of Li—O—Li bond.

## 3 Conclusions

This investigation found that very rapid water quenching of samples resulted in structurally uniform and chemically homogenous crystallites. The oven cooling of samples caused phase segregation of the crystallites resulting in a nickel rich surface phase, a manganese rich bulk phase, as well as intermediate transitional phases. We suggest here that the mechanism causing these differences is the extreme difference in cooling rate. The rapid cooling seen for the WQ sample increased the stabilization of the meta‐stable R‐3m phase, resulting in a more chemical homogenous and mechanically uniform sample. While the slow cooling of the OC sample was gradual enough the samples reached thermodynamic equilibrium and saw the formation of the two thermodynamically favorable phases, leading to defect formation and eventually phase segregation.

The increased homogeneity of the WQ resulted in higher performance materials as the more uniform distribution of ions caused a more uniform lattice strain during electrochemical cycling. This uniform distribution made the layered structure of the WQ material more resilient to defects, particularly antisite defects, allowing for the gradual formation of Li—O—Li bonds over the course of cycling, which can help explain the gradual increase in capacity seen in the WQ samples. In contrast, the phase segregation seen in the OC sample allowed for the buildup defects in the Ni‐rich surface phase, and lattice stress on the surface/bulk interface, both of which expedited surface dissolution and materials degradation.

## 4 Experimental Section

### Materials Synthesis

Cathodes materials were prepared following the sol–gel methods described by Park et al. following the stoichiometric ratios of Li[Ni*_x_* Li_1/3−2_ *_x_*_/3_Mn_2/3−_ *_x_*_/3_]O_2_. For this investigation *x* = 0.25, making the end products have the layered notation of Li[Ni_0.25_Li_0.167_Mn_0.583_]O_2_.^[^ ^38^ ^]^ The initial gel was prepared by dissolving stoichiometric amount of Mn(CH_3_COO)_2_·4H_2_O, Ni(NO_3_)_2_·6H_2_O, and Li(CH_3_COO)·2H_2_O (with 5% excess) in 100 °C water until gelation. This gel was fired at 400 °C in an alumina crucible, ground by mortar and pestle, fired at 500 °C, reground, then finally fired at 900 °C for 24 h. For the water quenched sample, the crucible was inverted over an excess of ambient temperature deionized water to quench the sample, before being dried in a 70 °C ambient oven overnight; this process is consistent with the initial publication.^[^ ^1^ ^]^ For the oven cooled material, the oven was stopped and the oven, with the crucible in it, was allowed to naturally cool to ambient temperature. All firing was conducted in ambient atmosphere in a box furnace. For all samples, there was no noticeable residual salts on the material powders or in the quench water. For estimations of the cooling rate experienced by the samples please refer to the Supporting Information 1.

### TEM/STEM/EDS

Transmission electron microscopy (TEM), scanning tunneling electron microscopy (STEM), and energy dispersive X‐ray spectroscopy (EDS) were conducted on a ThermoFisher Themis 200 G3, equipped with a X‐FEG electron source, a Cs DCOR probe corrector, and a Super‐X EDS detector. EDS was collected in High‐Angle Annular Dark Field (HAADF) with a voltage of 200 kV, a 50 µs dwell time, with collection occurring for 15 min. Sample powders were suspended in ethanol and then deposited onto lacey carbon/copper TEM grids purchased from Ted Pella, these grids where then dried in a 70 °C vacuum oven before use.

### XPS

XPS  was conducted using a cylindrical Mirror analyzer (DESA 100 Energy Analyzer developed by Staib, GMB). An Al K*α* (1486.6 eV) anode was used as the X‐ray emission source. XPS analysis was carried to determine the binding energies of nickel, manganese, and oxygen on the surface of the fully cast cathodes. The binding energies of the materials were deconvoluted using a mixture of the CasaXPS, Fityk, and Matlab software packages. Binding energy was calibrated for each sample using the adventurous carbon peak.

### XRD

X‐ray diffraction was conducted on a Malvern Panalytical Empyrean XRD between 2*θ* values of 15° and 75° using Cu K_*α*_ radiation. Peak ratios were done with intensity values taken from a Highscore Plus background subtracted peak lists. The XRD patterns of postcycling samples in Figures 3 and 4 were corrected for instrumental displacement, and low angle scattering stems from amorphous carbon present in the samples.

### Coin Cell Assembly

Cathode powders were mixed with Super‐P carbon black and polyvinylidene fluoride (PVDF) in a mass ratio of 8:1.5:0.5 in *N*‐Methyl‐2‐Pyrrolidone. First PVDF was added to 80 °C NMP, then carbon black was added to the mixture, then the solution temperature was increased to 100 °C and the active materials were added to the mixture, there was roughly an hour between additions. The resultant slurry was then sonicated before being spray coated onto 200 °C 10 µm thick aluminum foil. This coated foil was then dried at 70 °C in ambient atmosphere overnight. 12 mm diameter circular sections of coated foil were then used in 2032 coin cells with a lithium metal anode, 1.0 m LiPF_6_ 50/50 EC:DMC electrolyte, celgard battery separator, 0.5 mm stainless steel spacer, and wave spring. Each coil cell was sealed with an MTI coin cell press in a dry, low oxygen argon atmosphere. A minimum of 3 coin cells was made and cycled with data coming from the representative cells.

### Electrochemical Characterization

Coin cells in Figure 2 underwent constant current potential limited galvanostatic testing on a LANHE battery tester with a maximum current rating of 1 mA and a resolution of 0.001 µA. Cells were cycled between the potentials of 2.0 and 4.8 V. Two formation cycles were performed at C/20 rate for both charge and discharge. This was followed by 25 cycles with C/20 charges and C/2 discharges, then two symmetric C/20 cycles, this was repeated once more ending on the final C/20 symmetric cycles for a total of 56 cycles. The cells were cycled in a room with an average temperature of 20 ±2 °C, with these temperature changes likely accounting for the fluctuations seen in the capacity of the coin cells.

Potentials in this investigation are measured versus the Li/Li^+^ pseudoreference, which has a potential of −3.05 V compared to the standard hydrogen electrode.

## Conflict of Interest

The authors declare no conflict of interest.

## Supporting information

Supporting Information

## Data Availability

The data that support the findings of this study are available from the corresponding author upon reasonable request.
