# Supplementary material for: The Importance of Structural Uniformity and Chemical Homogeneity in Cobalt‐Free Lithium Excess Nickel Manganese Oxide Cathodes
Source: Adv Sci (Weinh). 2023 Apr 17;10(16):2300068. doi: 10.1002/advs.202300068 (PMC10238218; doi:10.1002/advs.202300068)
Supplement: Supplementary file 1 — Supporting Information [file ADVS-10-2300068-s001.pdf]

ADVANCED SCIENCE
Open Access

Supporting Information

for *Adv. Sci.*, DOI 10.1002/advs.202300068

The Importance of Structural Uniformity and Chemical Homogeneity in Cobalt-Free Lithium Excess Nickel Manganese Oxide Cathodes

*Sven Burke and Jay F. Whitacre**

Supplemental Information

SI1 – This image demonstrates the cooling rate of the water quenched samples. The thermal camera used had a frame rate of 6 frames a second, and has a lower bound of 100 °C. The camera record the material going from 900 °C to 100 °C over the course of 4/6 of a second, so 800°C /(2/3 sec) = 1200 °C/sec, is the slowest cooling rate of the materials. By comparison the oven cooled sample took roughly 3 hours to go from 900 °C to 22 °C, so the 878 °C/ (10,800 sec) = 0.0813 °C/sec, is the approximate cooling rate of the oven cooled sample. The difference between these two rates is then 1200/0.0813 = 14,760, or a difference of 5 orders of magnitude.

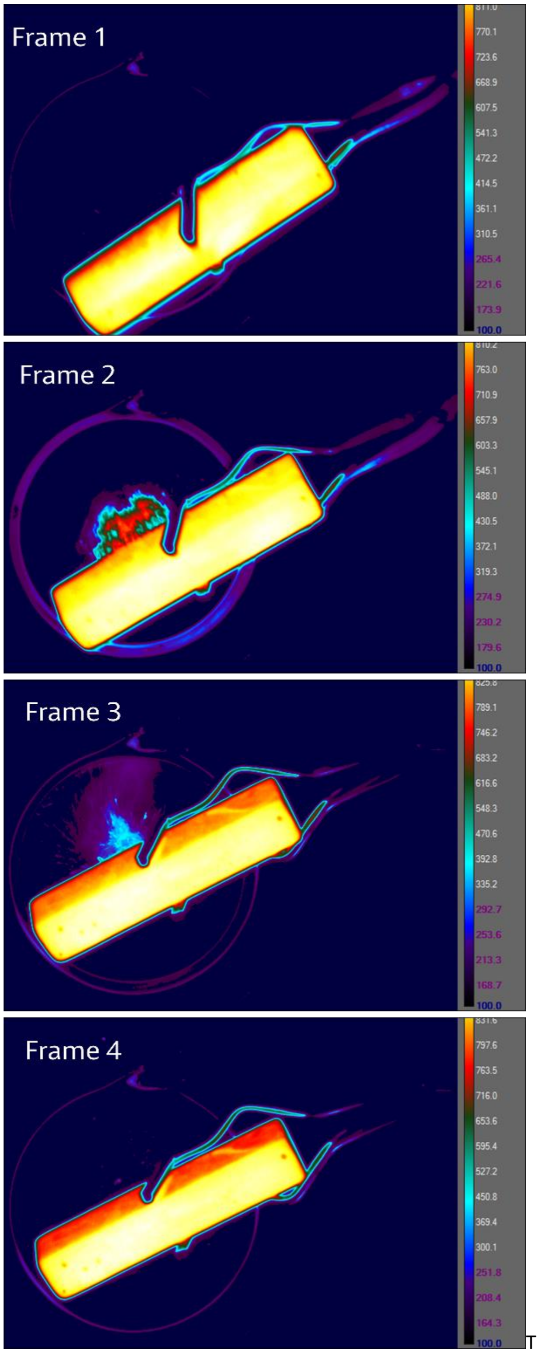

T

SI2 – This table shows the XRD peak ratios of the WQ-pre and OC-pre samples.

**Sample**	**Peak Intensity Ratios**	
	**$I_{(003)}/I_{(104)}$**	**$(I_{(006)}+I_{(102)})/I_{(101)}$**
**WQ-pre**	***1.56 ± 0.020***	***0.30 ± 0.003***
**OC-pre**	***1.48 ± 0.019***	***0.44 ± 0.004***

The **$I_{(003)}/I_{(104)}$** ratio is used to determine the relative amount of Li^{+}/Ni^{2+} anti-site defects in the sample, with the higher ratio indicating less anti-site defects and therefore more inter-layer order [1].

The **$(I_{(006)}+I_{(102)})/I_{(101)}$** ratio is used to determine the intra-layer ordering of the sample, with the lower ratio indicating more of a hexagonal arrangement and therefore more entropic and disordered system [1–3].

SI3 – The color comparison of the samples. a) the oven cooled powder next to a block of color that is the average color of the powder and has the RGB value of the average color provided, b) the water quenched powder next to a block of color that is the average color of the powder and has the RGB value of the average color provided, c) the UV-Vis spectra of suspensions of both samples in water at the same concentration of powder to H_2O, d) a zoom in on the 380 – 450 nm section of the spectra from c) that highlights the differnece between the two samples. The average color values of the powders was collected through taking the color value of 10 pixels form each powder at random and averaging the values. The UV-Vis spectra were done through suspending 5 mg of powder in 5 ml of DI water then sonicating, a 2 ml cuvette was then filled with DI water and 30 µL of the previous suspension, the cuvettes were inverted and sonicated before spectra were collected

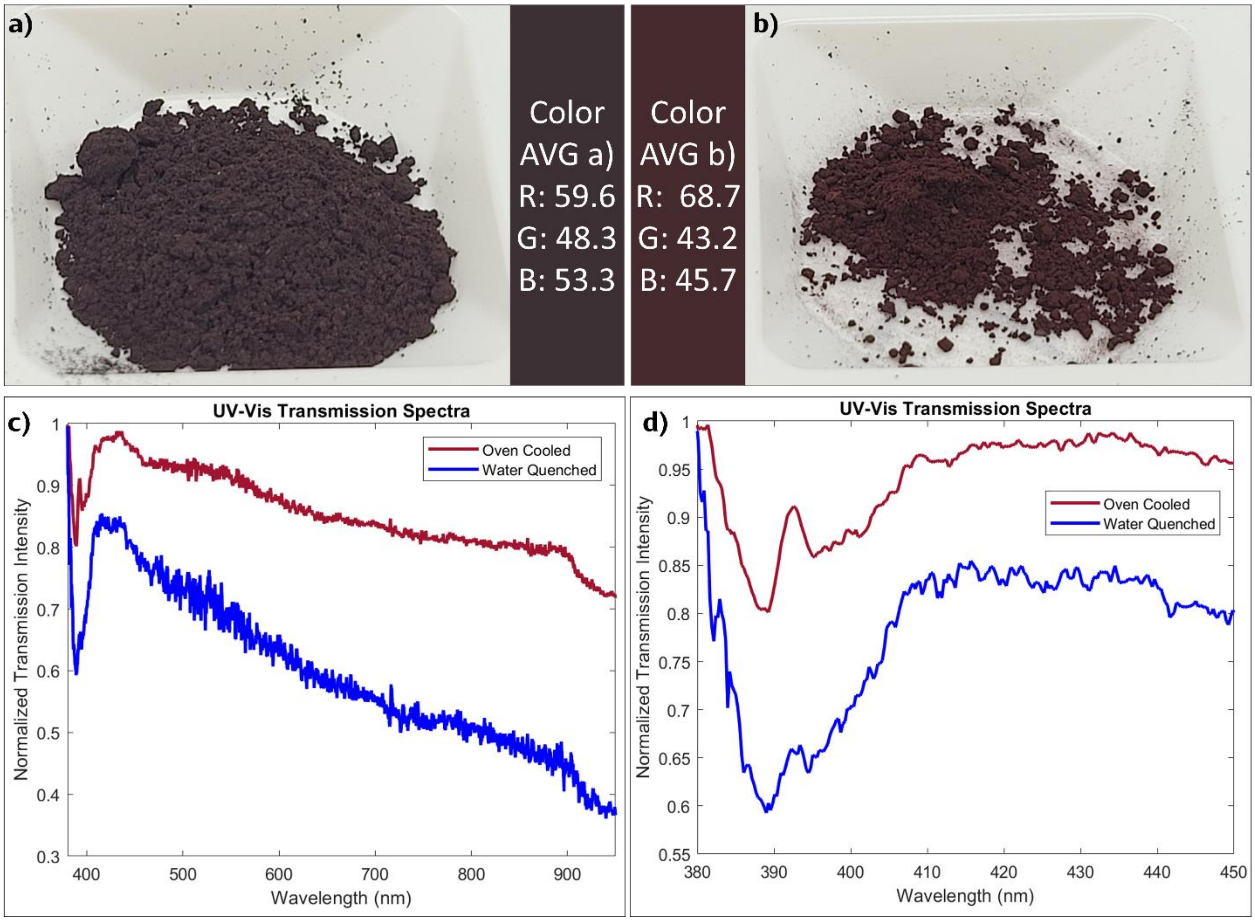

SI4 – These particles were used for the STEM and EDS micrographs seen in figure 1 b) – e), and are imaged along the same zone-axes and are similar size and shape. a) A TEM image of the WQ-pre particle, some carbon was found to have deposited over the course EDS imaging, b) A STEM image of the OC-pre particle, c) The SAED pattern for the WQ-pre sample particle, indexed to (241) with two super lattice reflections, d) The SAED pattern for the OC-pre sample particle, indexed to (241) with two super lattice reflections.

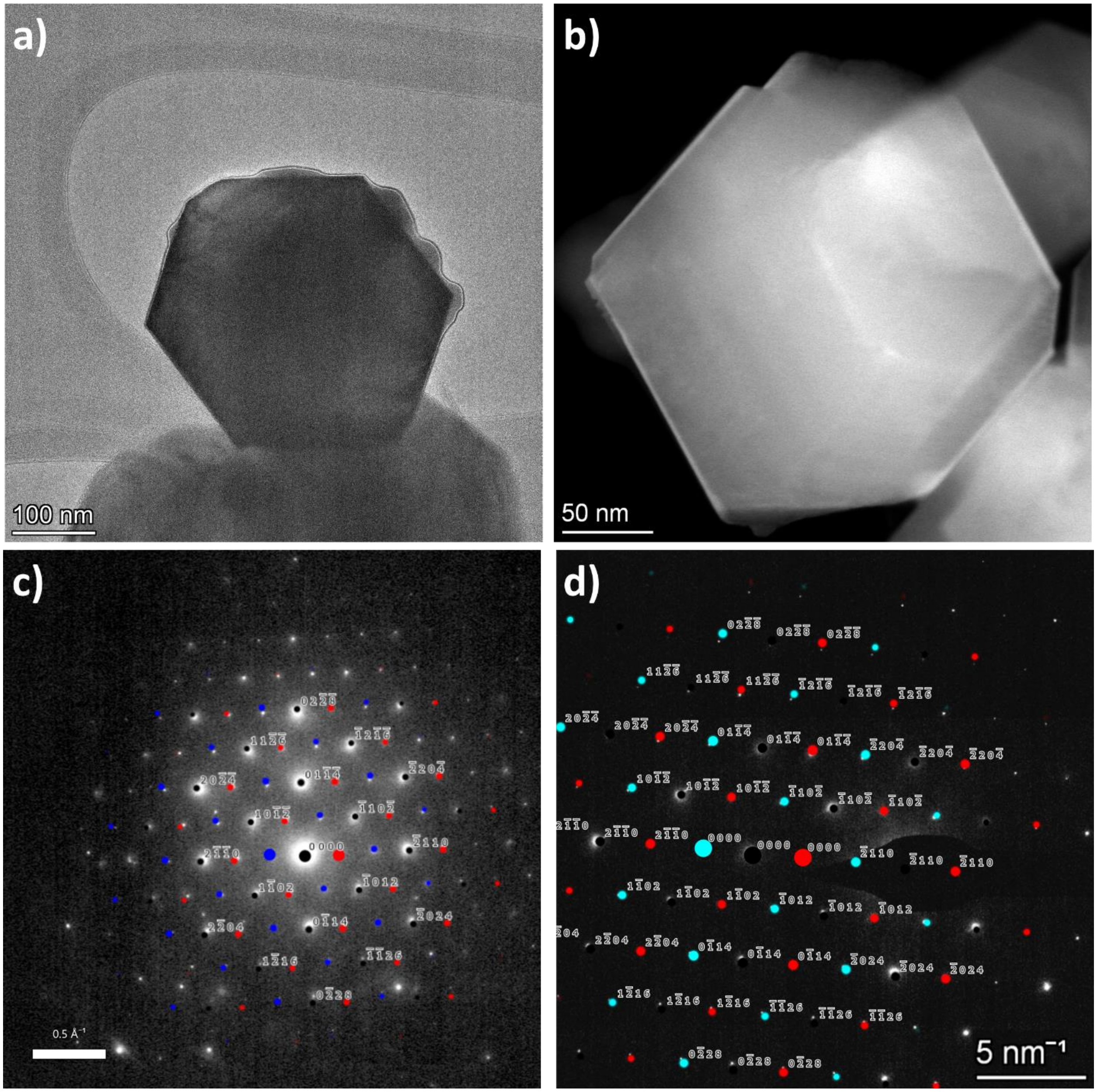

SI5 – STEM micrograph of the WQ-pre sample with the FFT patterns of each region of the particle.

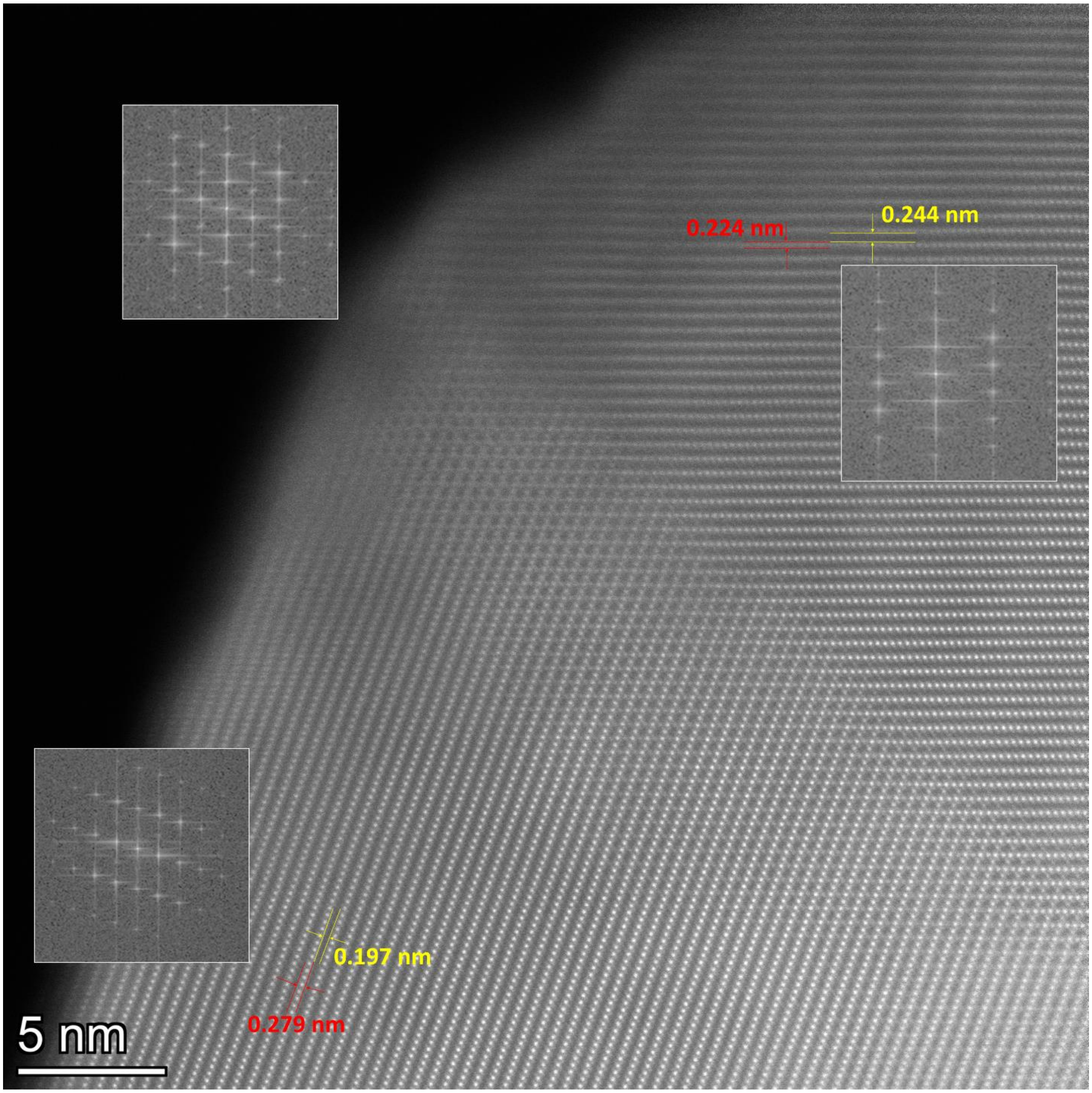

SI6 – STEM micrograph of the OC-pre sample with the FFT patterns of each region of the particle. The red line denotes a different focus plane and where the resultant images were stitched together.

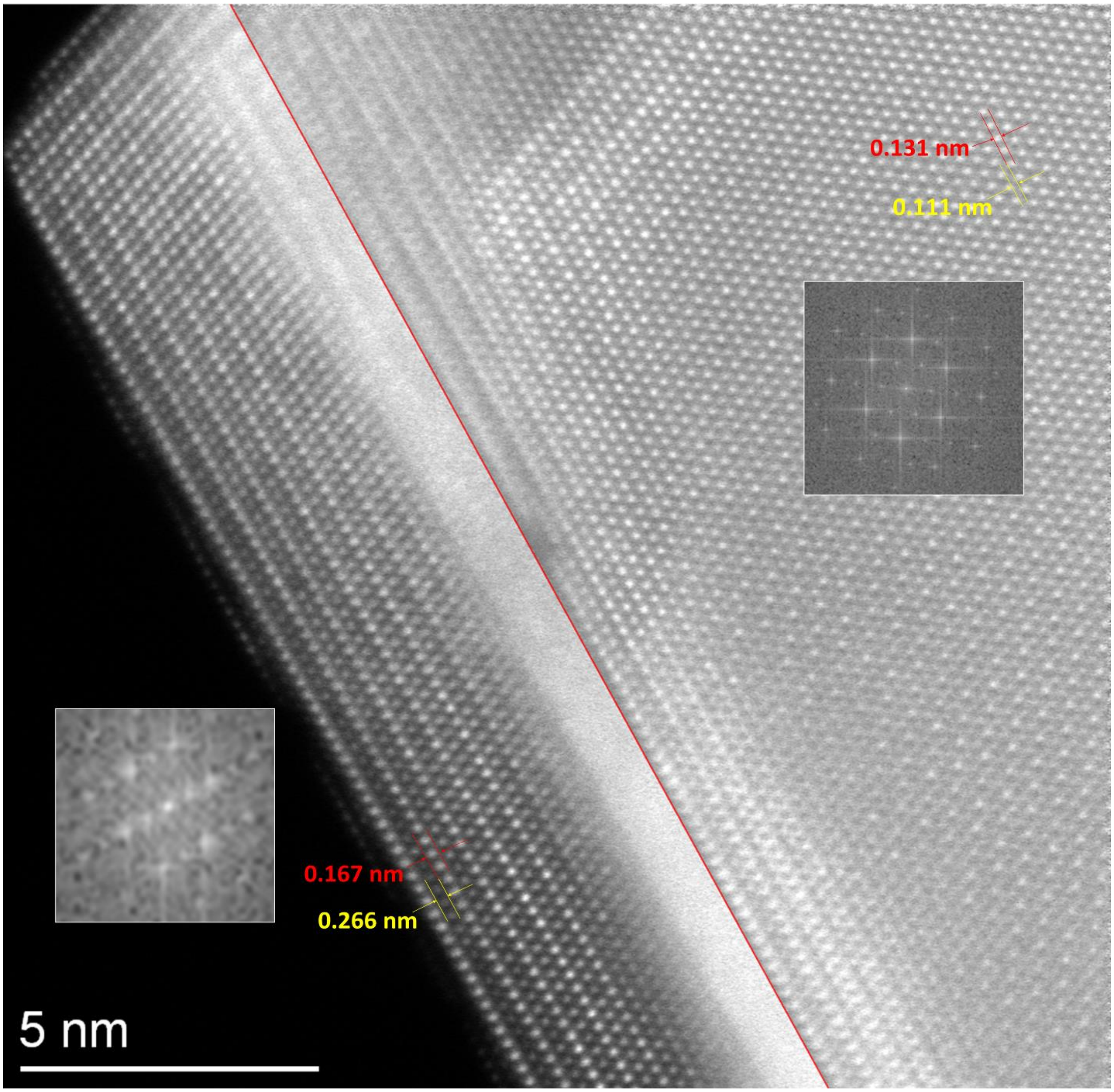

SI7: The specific discharge capacities and capacity averaged discharge voltage of each cycle for both WQ and OC samples from figure 2.

	Specific Discharge Capacity (mAh/g)		Average Discharge Voltage (V vs Li/Li^{+})	
Cycle Number	Sample WQ	Sample OC	Sample WQ	Sample OC
1	155.8	196.0	3.483	3.694
2	164.5	195.3	3.561	3.682
3	147.7	163.1	3.409	3.600
4	153.6	159.7	3.410	3.587
5	148.0	154.0	3.403	3.580
6	160.1	155.8	3.423	3.585
7	164.9	159.9	3.407	3.575
8	159.4	153.9	3.383	3.564
9	157.3	154.9	3.386	3.560
10	166.3	160.6	3.406	3.564
11	172.6	157.8	3.396	3.554
12	170.3	152.1	3.387	3.545
13	164.8	155.1	3.379	3.548
14	167.8	157.9	3.376	3.535
15	173.5	150.8	3.381	3.509
16	174.3	153.2	3.371	3.508
17	171.7	157.6	3.360	3.512
18	163.2	155.3	3.335	3.484
19	165.8	153.9	3.354	3.477
20	172.6	156.9	3.346	3.481
21	174.2	157.4	3.342	3.468
22	171.4	153.3	3.320	3.443
23	166.4	154.6	3.331	3.433
24	168.8	157.2	3.312	3.433
25	176.2	153.0	3.323	3.407
26	175.8	156.2	3.315	3.404
27	165.4	157.6	3.289	3.405
28	230.7	188.9	3.416	3.565
29	232.7	192.1	3.435	3.564
30	172.3	159.2	3.289	3.381
31	177.7	156.9	3.297	3.353
32	178.9	153.2	3.284	3.332
33	175.1	157.8	3.268	3.344
34	171.0	158.3	3.259	3.341
35	171.8	153.9	3.252	3.301
36	179.0	155.5	3.261	3.312
37	178.3	157.6	3.255	3.315
38	175.8	156.0	3.237	3.288

39	165.7	155.1	3.213	3.285
40	170.1	156.0	3.218	3.285
41	178.7	156.9	3.232	3.276
42	179.1	151.0	3.224	3.234
43	178.9	154.4	3.211	3.247
44	176.8	155.8	3.198	3.258
45	177.8	149.6	3.203	3.212
46	176.3	152.1	3.188	3.220
47	182.5	156.5	3.195	3.243
48	183.1	154.6	3.191	3.223
49	181.3	152.4	3.174	3.209
50	175.8	154.9	3.170	3.238
51	178.8	156.7	3.182	3.229
52	182.1	151.0	3.176	3.182
53	188.3	151.9	3.172	3.187
54	187.1	155.5	3.164	3.205
55	251.8	191.6	3.323	3.477
56	252.3	187.7	3.329	3.488

SI8 – This table provides the XRD peak ratios of the WQ-post and OC-post samples. These ratios are used to determine the concentration of anti-site defects, $I_{(003)}/I_{(104)}$, and the degree of order in the transition metal layer, $(I_{(006)}+I_{(102)})/I_{(101)}$, of the samples.

**Sample**	**Peak Intensity Ratios**	
	**$I_{(003)}/I_{(104)}$**	**$(I_{(006)}+I_{(102)})/I_{(101)}$**
**WQ-post**	***1.26 ± 0.016***	***0.24 ± 0.002***
**OC-post**	***1.19 ± 0.015***	***0.34 ± 0.003***

SI9 – a) A STEM image of the WQ-post particle, b) A TEM image of the OC-post particle, c) The SAED pattern for the WQ-post sample particle, indexed to (-110) with four super lattice reflections, d) The SAED pattern for the OC-post sample particle, indexed to (241) with four super lattice reflections.

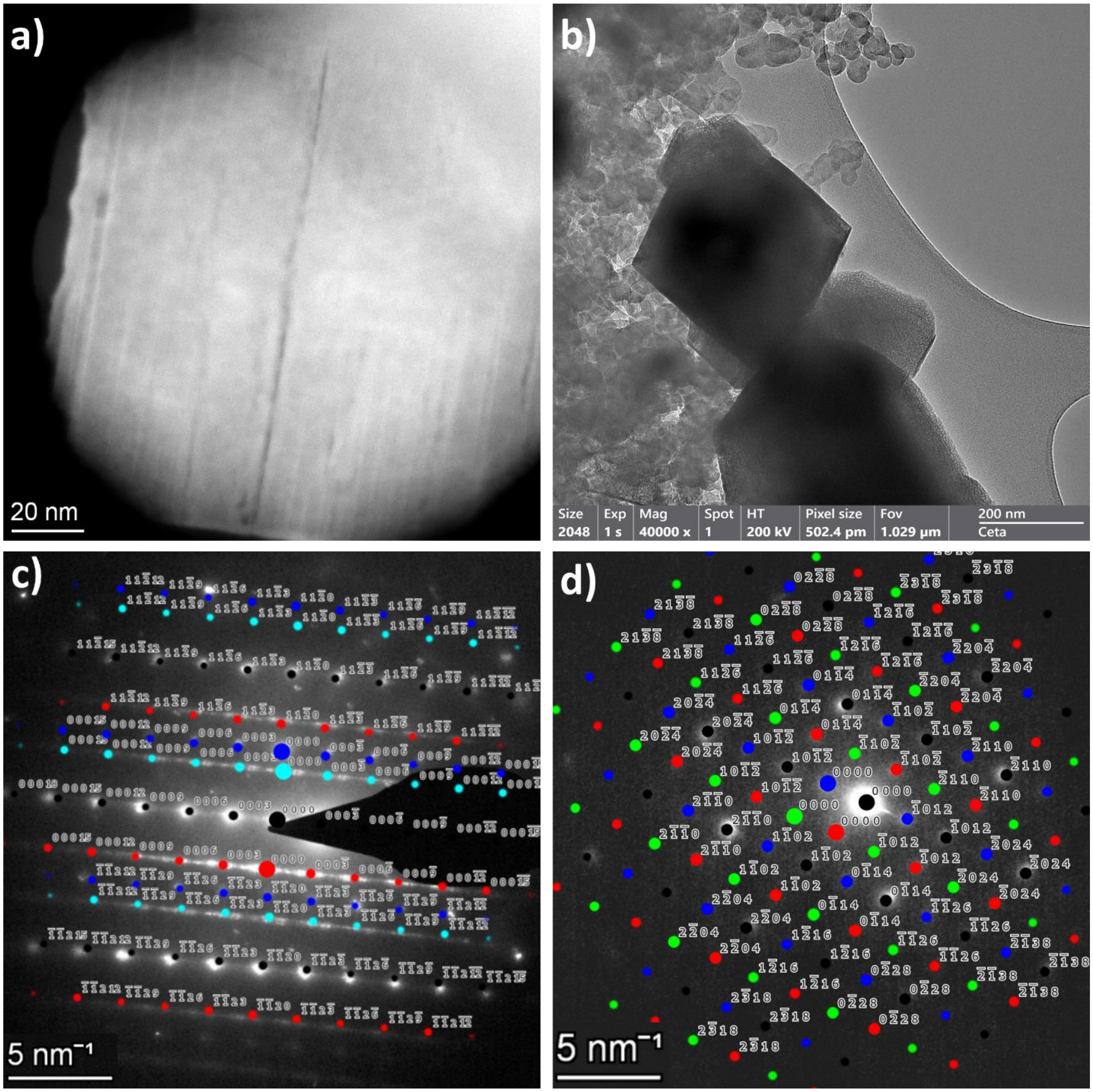

It is important to note that the SAED patterns shown for the WQ-post and OC-post samples have 3 reflections, while the SAED patterns of WQ-pre and OC-pre only have two. These increases in diffraction reflections demonstrates that the transition metals in the sample have migrated over the course of cycling and are likely more dispersed.

SI10 – STEM micrograph of the WQ-post sample with the FFT patterns of each region of the particle.

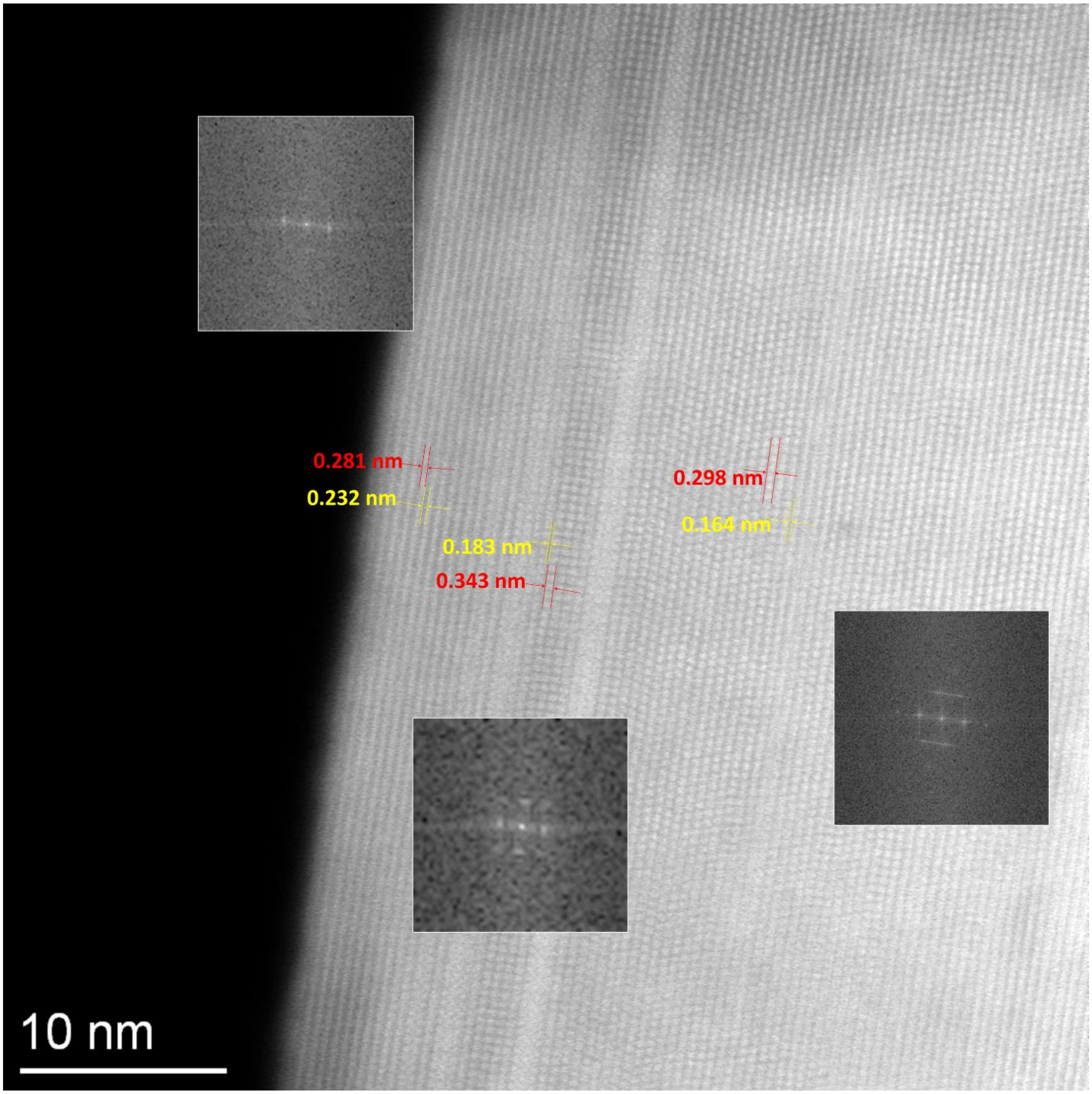

SI11 – STEM micrograph of the OC-post sample with the FFT patterns of each region of the particle.

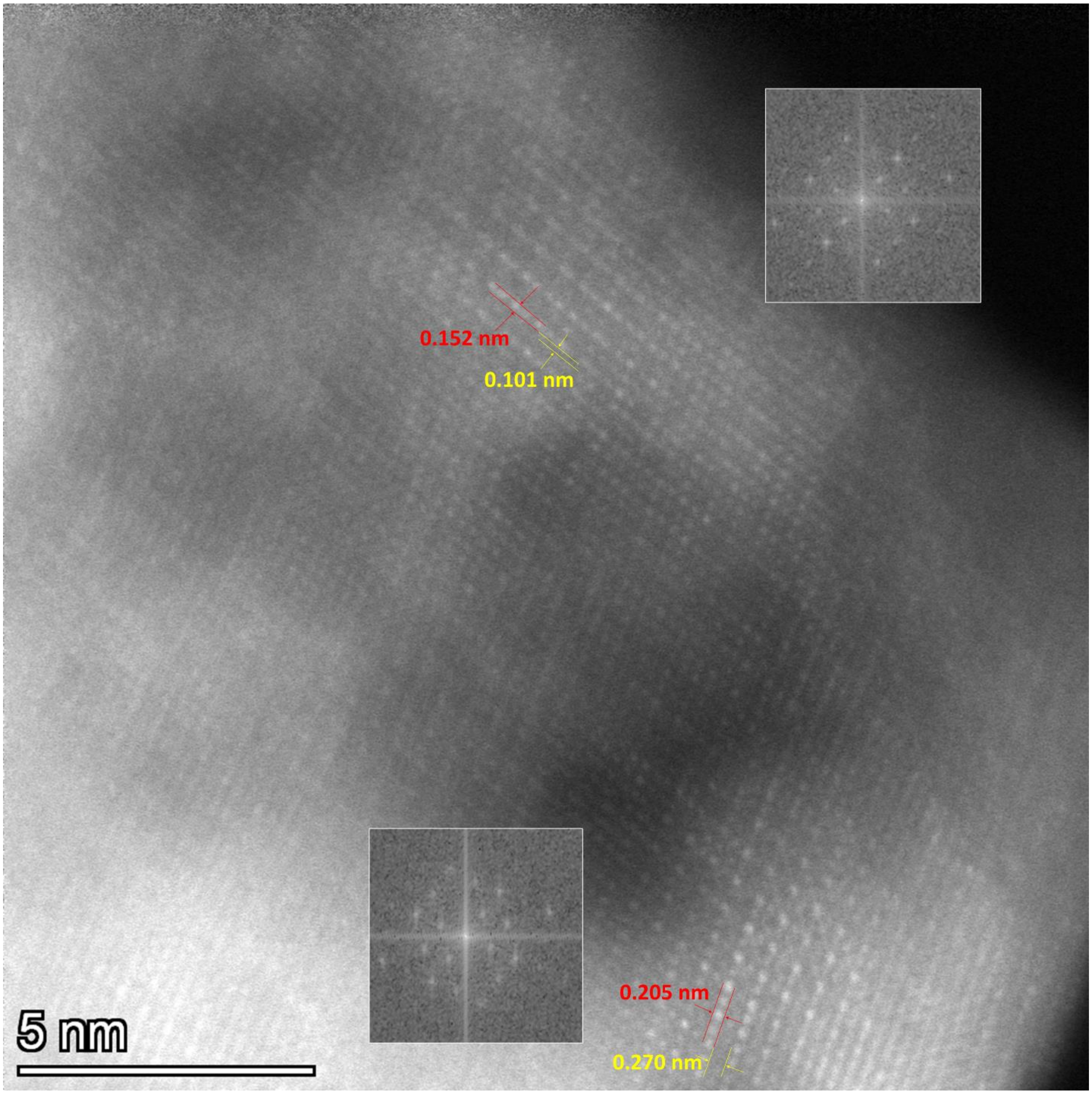

SI12 – a) STEM micrograph of the OC-post sample spinel region found nucleating on the side of the main particle, with the 3 regions and their FFT Patterns shown, b) the EDS map of the region with 3 regions used shown, c) the table of EDS results for the sample.

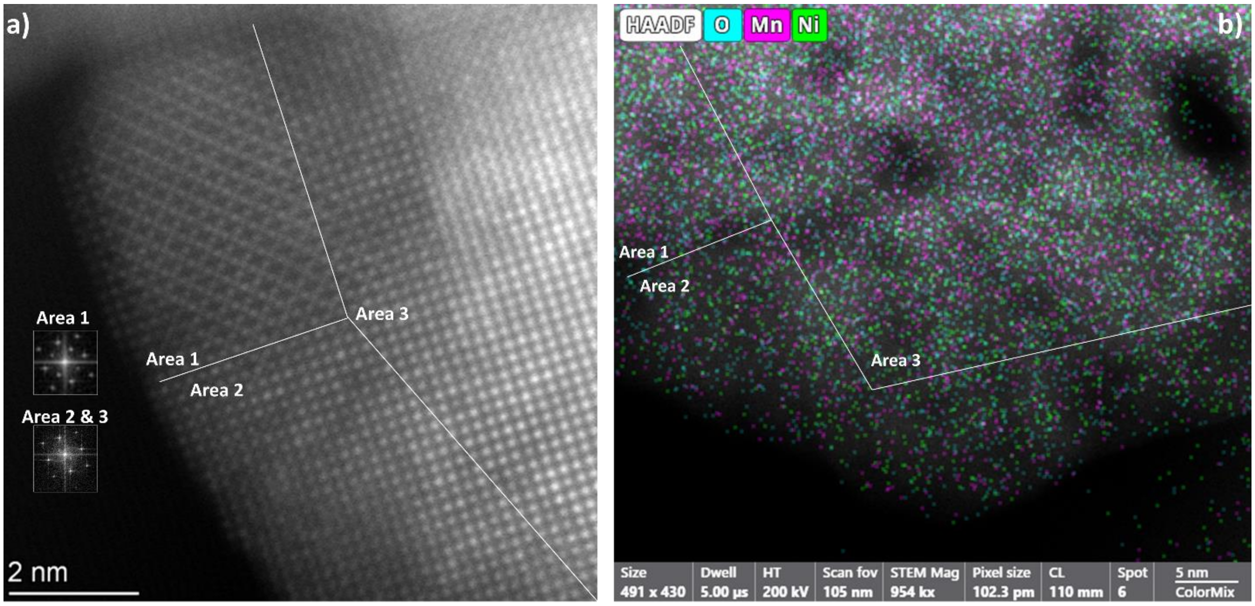

c)

Spinel Area					
**Area**	**Mn at%**	**Ni at%**	**O at%**	**Mn/Ni**	**O/(Mn+Ni)**
**1**	**16.89**	**15.31**	**67.8**	***1.10***	***2.11***
**2**	**19.61**	**21.3**	**59.08**	***0.92***	***1.44***
**3**	**26.89**	**15.03**	**58.08**	***1.79***	***1.39***
**Whole Image**	**26.9**	**14.64**	**58.46**	***1.84***	***1.41***

SI13 – The XPS spectra of Oxygen 2S states for the i) WQ-pre ii) OC-pre iii) WQ-post iv) OC-post samples.

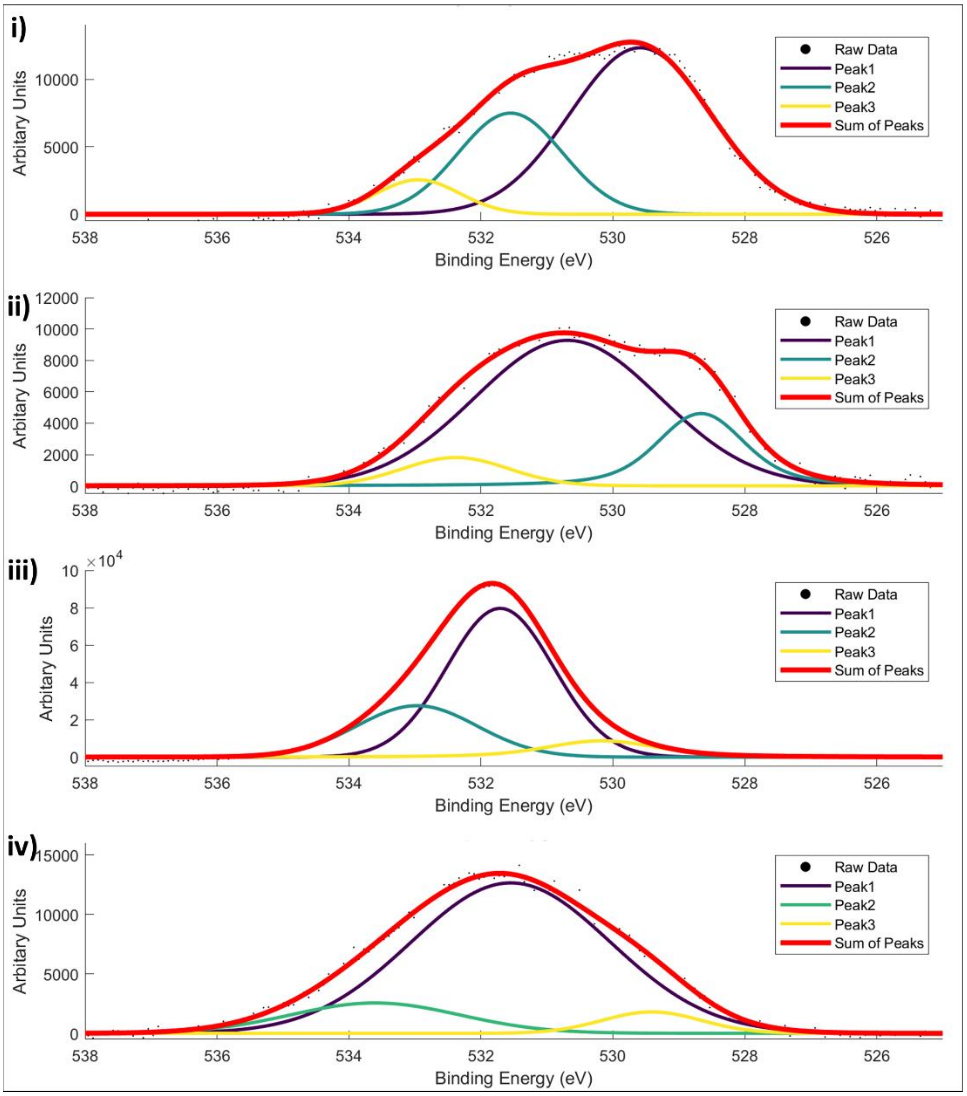

The oxygen 2s energies provided in SI12 demonstrate the most significant chemical differences between the WQ and OC samples. While both WQ-pre and OC-pre have peaks that indicate the presence of O-TM bonding, peaks 1 and 2 respectively, the lower energy of the OC-pre O-TM peak indicates the O-TM bond is longer in OC-pre than in WQ-pre, which would be consistent with a more nickel-rich surface. Additionally, the OC-pre sample's most prominent peak, peak1, corresponds to the oxygen vacancies, which might explain the low O/TM ratio seen in the OC-pre sample [4–8]. After cycling the predominance of the oxygen vacancy peaks becomes stronger for both samples, indicating the formation of more oxygen vacancies. While this is consistent with the reduced O/TM ratios seen for the WQ-post sample it is not consistent with the increased O/TM ratios seen in the OC-post sample. This would suggest that the OC-post sample has more oxygen vacancies than the OC-pre sample, and that the O/TM ratio is driven up by the dissolution of TM, and not the addition of oxygen into the lattice. Finally, the WQ-post XPS spectra sees the emergence of peak 3 at 530.38 eV, this higher oxygen energy is evidence of the formation of Li – O – Li cross layer bonds over the course of cycling. These Li – O – Li bonds are associated with higher energy in lithium excess cathode systems and the emergence of this peak from cycling indicates that cycling and the migration of transition metals associated with it caused

the formation of Li – O – Li bonds, which could account for the increasing capacity of the WQ sample during electrochemical cycling.

SI14 – The XPS spectra of Li 1s and Mn 3P states for the i) WQ-pre ii) OC-pre iii) WQ-post iv) OC-post samples.

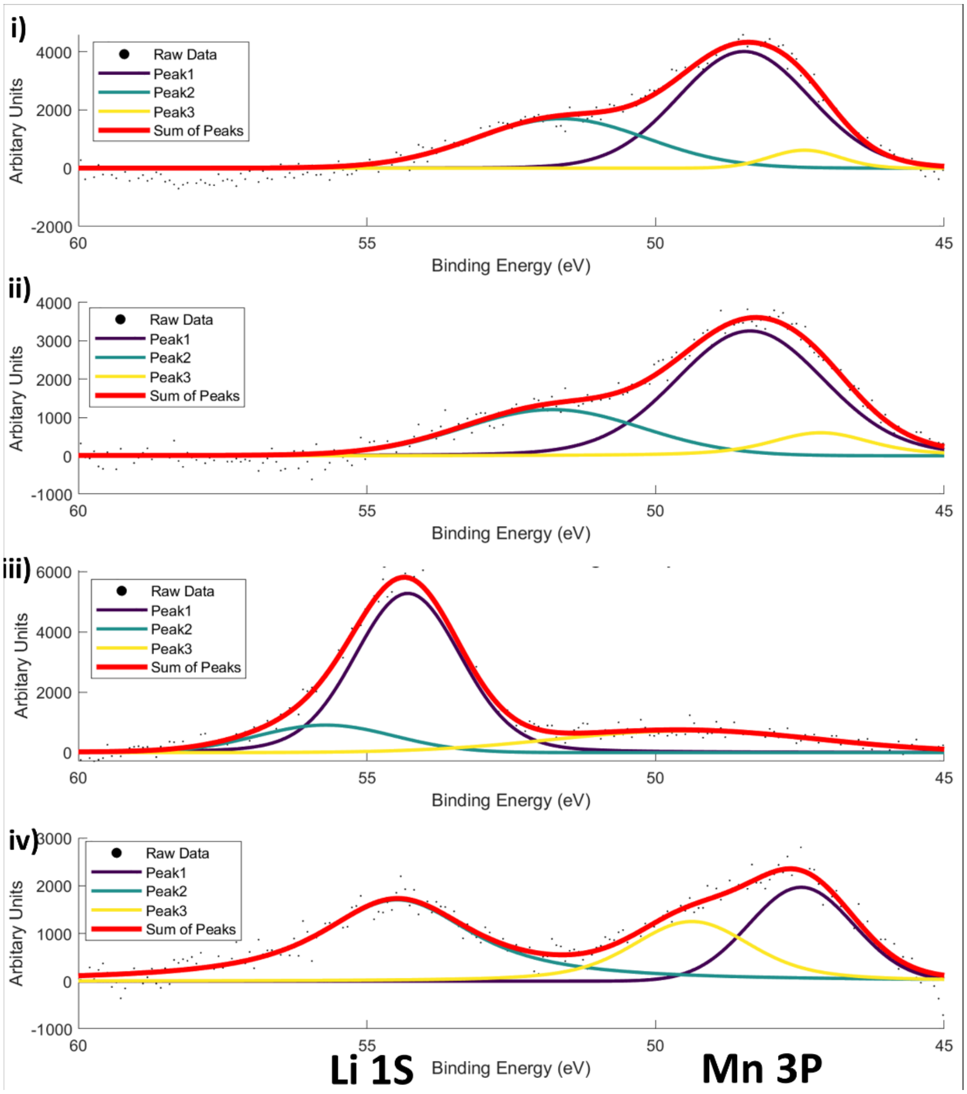

SI13 shows that the initial energy states of the WQ and OC samples for both Li 1s and Mn 3p states are almost identical, with no Li 1s signal and Mn 3p signal indicating the presence of Mn^{4+}. The data show that the WQ-post sample sees reduced Mn^{4+} signal and high lithium signal; this is likely the result of transition metal migration. The data also show that the OC-post sample sees a downshift in Mn 3s energy indicating the oxidation of Mn^{4+} to Mn^{2+}, and a lithium 1s peak [8–11]; the formation of Mn^{2+} would provide further support for the dissolution of transition metals in the OC sample. The Mn^{2+} state is highly soluble and is often the byproduct of the $2Mn^{3+} \rightarrow Mn^{2+} + Mn^{4+}$ dissolution reaction [12,13]. The lithium energies seen in the spectra of both samples confirm the presence of lithium in the samples and

provide evidence of electrochemical cycling, particularly XPS peak2 of the WQ-post sample, which is close in energy to Li_2CO_3, a common byproduct of electrochemical cycling [7]. The changes in the manganese states seen in these samples over the course of cycling is further evidence of how the WQ sample is more electrochemical stable than the OC sample.

SI15 – The XPS spectrums with fitted peaks of the Mn 3s states for samples i) WQ-pre ii) OC-pre iii) WQ-post iv) OC-post.

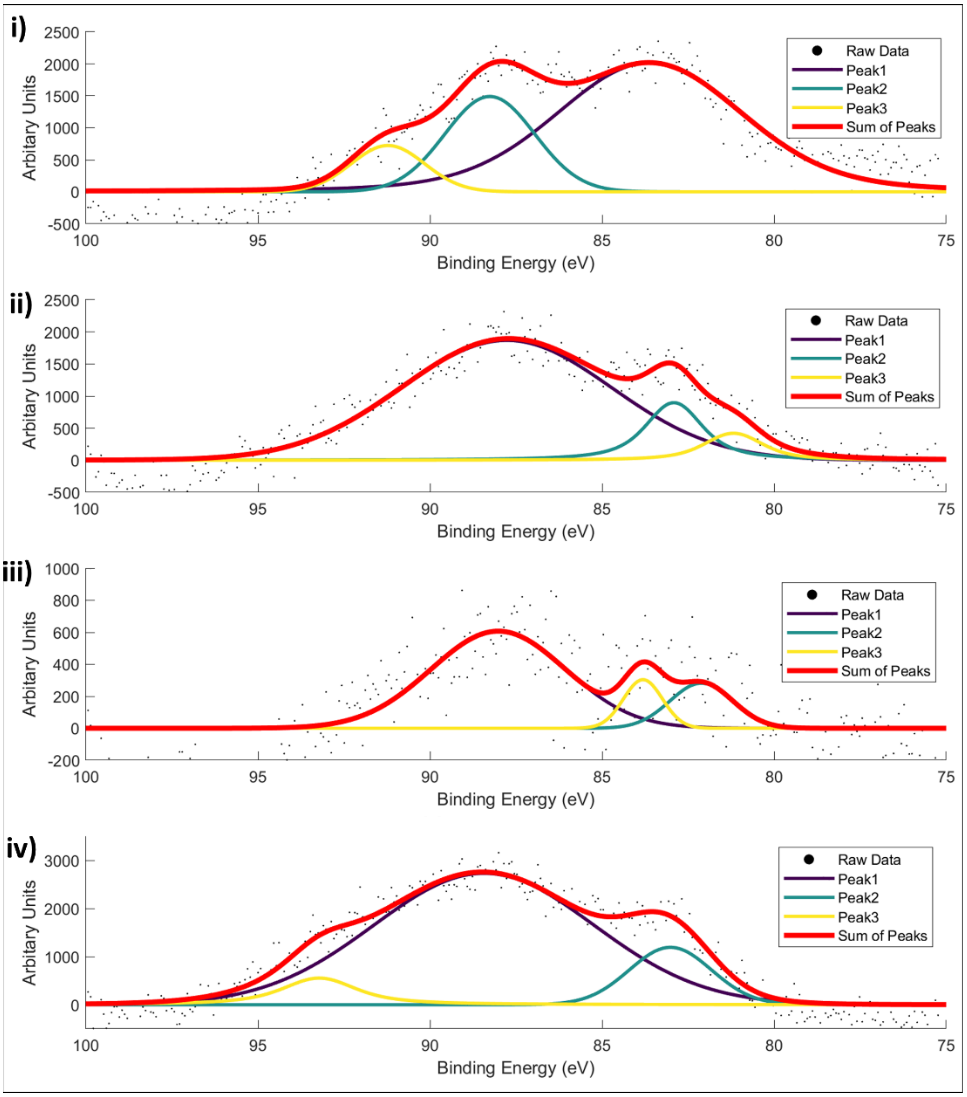

The Mn 3s peaks seen SI14 provide additional evidence of the transition metal dissolution occurring in the OC sample over the course of cycling. The difference in energy between the peaks 1 and 2 is roughly 4.64 eV, which is evidence of Mn^{4+} states, with the high energy peak3 providing further evidence of Mn^{4+}; the differences in the peak energies of OC-pre peaks 1 and 2 indicate the presence of Mn^{4+}, while the difference between peaks 1 and 3 is evidence of Mn^{2+}, which is highly soluble [71,72]. Cycling the samples demonstrates that some transition metal dissolution was occurring in both samples, with the energy differences between peaks 1 and 2 and peaks 1 and 3 of WQ-post indicating

the presence of Mn^{3+} and Mn^{4+} respectively, while the differences between the energies of peaks 1 and 2 in OC-post suggest the almost exclusive presence of Mn^{3+}, and the very high energy of the peak 3 suggesting an oxidation state of +3.5 [71,72]. The almost exclusive presence of Mn^{3+} energies in the XPS spectra of the OC-post sample's Mn 3s spectra is consistent with EDS and STEM evidence of Li_2MnO_3 like phases. As previously discussed, the relative abundance of this Mn^{3+} in this sample would also make this sample more susceptible to transition metal dissolution.

SI16 – The XPS spectrums with fitted peaks of the Mn 2P states for samples i) WQ-pre ii) OC-pre iii) WQ-post iv) OC-post.

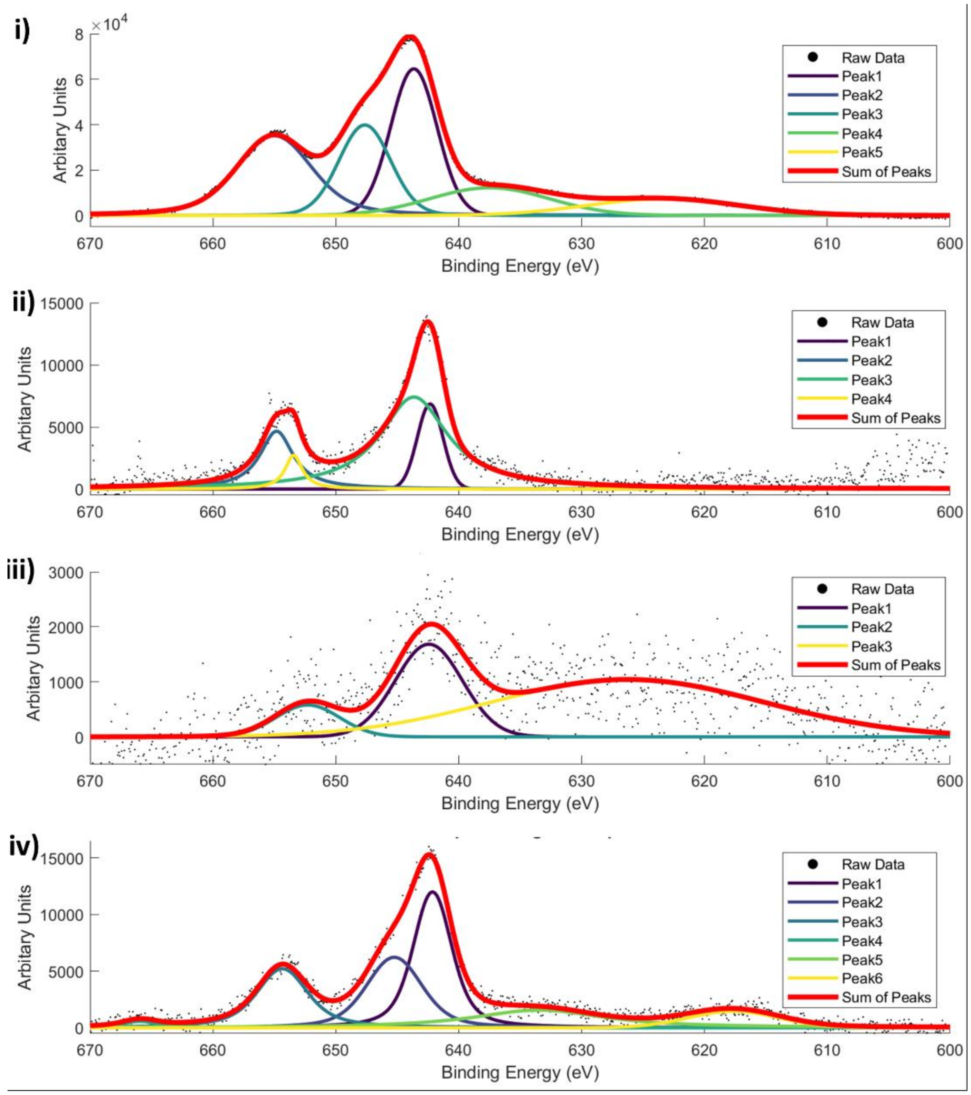

SI15 provides further evidence of the presence of these highly soluble oxidation states of manganese in the OC samples. The greater degree of lower manganese oxidation states might also help explain the more rapid voltage losses seen in the sample over the course of cycling. The Mn^{2+} and Mn^{3+} states are also Jahn-Teller active, which would only contribute to the lattice strain of the sample [62,69].

SI17 – The XPS spectrums with fitted peaks of the Ni 2P states for samples i) WQ-pre ii) OC-pre iii) WQ-post iv) OC-post.

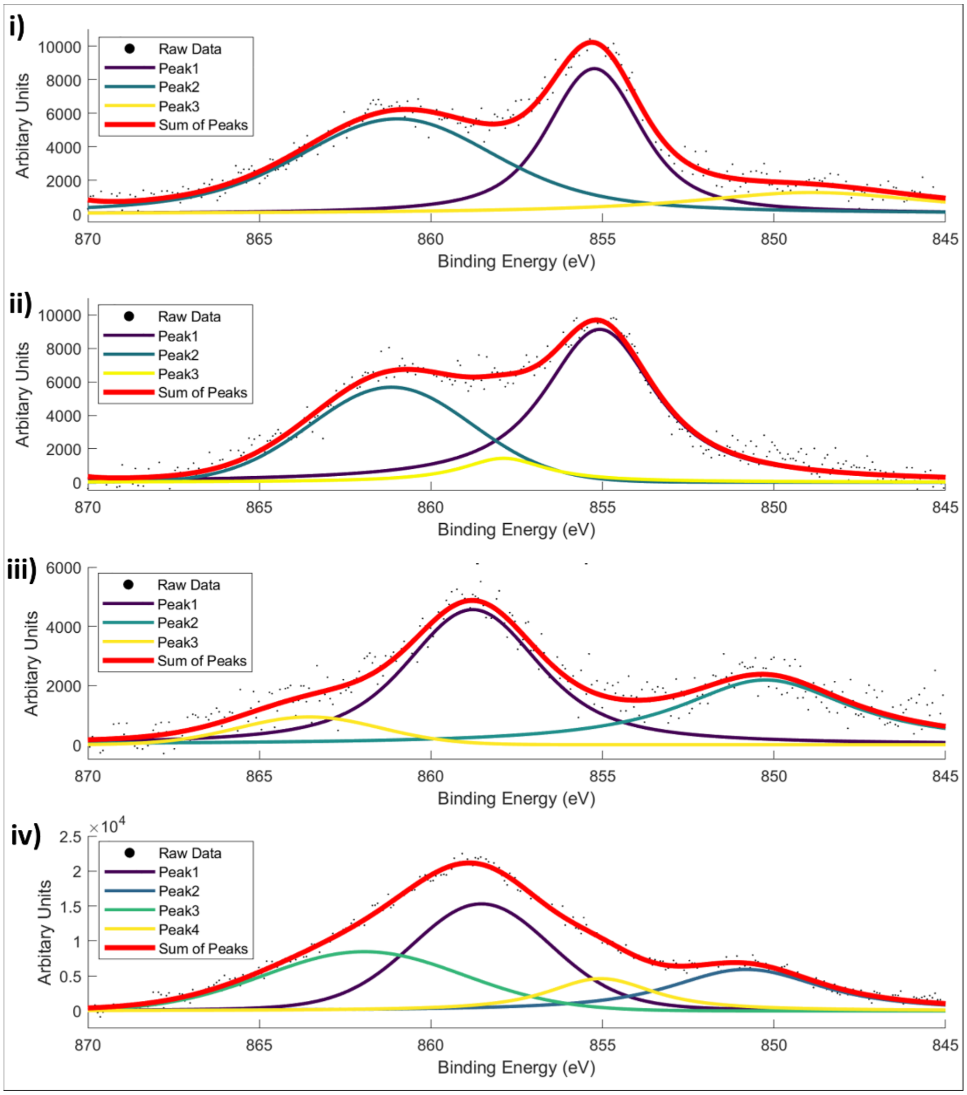

The nickel oxidation states for the samples in SI16 also provide additional evidence of OC samples experiencing Jahn-Teller effects, as in OC samples saw energy states that indicate the presence of the Ni^{3+}, another Jahn-Teller active transition metal ion.

SI18 – XPS peak position and shift tables, the tables below list the peak centers and R^2 values for the XPS fits of their respective ionic state.

Calibration Shifts				
WQ-pre			OC-pre	
carbon location	shift		carbon location	shift
284.84	-0.04		284.73	0.07
WQ-post			OC-post	
carbon location	shift		carbon location	shift
284.59	0.21		284.82	-0.02

Li 1s and Mn 3p								
WQ-pre					OC-pre			
R^2	Li 1s and Mn 3p				R^2	Li 1s and Mn 3p		
0.9721	peak 1	peak2	peak3		0.9762	peak 1	peak2	peak3
Center (eV)	48.43	51.59	47.38		Center (eV)	48.43	51.85	47.21
WQ-post					OC-post			
R^2	Li 1s and Mn 3p				R^2	Li 1s and Mn 3p		
0.9886	peak 1	peak2	peak3		0.9220	peak 1	peak2	peak3
Center (eV)	54.5	55.93	49.7		Center (eV)	47.46	54.46	49.35

O 1S								
WQ-pre					OC-pre			
R^2	Oxygen 1s				R^2	Oxygen 1s		
0.9900	peak 1	peak2	peak3		0.9945	peak 1	peak2	peak3
Center (eV)	529.54	531.51	532.91		Center (eV)	530.75	528.74	532.45
WQ-post					OC-post			
R^2	Oxygen 1s				R^2	Oxygen 1s		
0.9986	peak 1	peak2	peak3		0.9964	peak 1	peak2	peak3
Center (eV)	531.91	533.18	530.38		Center (eV)	531.53	533.6	529.39

Mn 3s								
WQ-pre					OC-pre			
R^2	Mn 3s				R^2	Mn 3s		
0.8738	peak 1	peak2	peak3		0.9018	peak 1	peak2	peak3
Center (eV)	83.57	88.22	91.18		Center (eV)	87.84	82.98	81.25
WQ-post					OC-post			
R^2	Mn 3s				R^2	Mn 3s		
0.4629	peak 1	peak2	peak3		0.9480	peak 1	peak2	peak3
Center (eV)	88.22746	82.32652	84.02236		Center (eV)	88.41411	82.99432	93.19958

Ni 2P													
WQ-pre							OC-pre						
R^2	Ni 2P						R^2	Ni 2P					
0.9629	peak 1	peak2	peak3	peak4	peak5		0.9622	peak 1	peak2	peak3	peak4	peak5	peak6
Center (eV)	855.20	860.93	872.82	879.21	848.87		Center (eV)	855.15	861.22	881.10	878.12	857.95	873.04
WQ-post							OC-post						
R^2	Ni 2P						R^2	Ni 2p					
0.8906	peak 1	peak2	peak3				0.9917	peak 1	peak2	peak3	peak4		
Center (eV)	858.99	850.46	863.76				Center (eV)	858.51	850.82	861.94	855.05		

Mn 2p													
WQ-pre							OC-pre						
R^2	Mn 2p						R^2	Mn 2p					
0.9980	peak 1	peak2	peak3	peak4	peak5		0.9702	peak 1	peak2	peak3	peak4		
Center (eV)	643.60	654.97	647.60	637.45	623.37		Center (eV)	642.39	654.89	643.72	653.50		
WQ-post							OC-post						
R^2	Mn 2p						R^2	Mn 2p					
0.5761	peak 1	peak2	peak3				0.9817	peak 1	peak2	peak3	peak4	peak5	peak6
Center (eV)	642.64	652.49	626.47				Center (eV)	642.11	645.22	654.33	665.93	633.46	617.62

SI19 – Refinement of XRD patterns

Sample	Rwp	lattice a (Å)	lattice c (Å)	point group	particle size (μm)	anti-site defects
**WQ-pre**	4.509	2.853	14.291	R-3m	0.0978	0.0478
**WQ-post**	8.384	2.858	14.293	R-3m	0.0552	0.0065
**OC-pre***	15.800	2.863	14.255	R-3m	0.0684	0.0338
**OC-post**	7.823	2.878	14.378	R-3m	0.0531	0.0465

* The OC-pre refinements never achieved R_{wp} lower than 15.8 despite undergoing the same refinement processes as the other samples. Additional attempts at two phase refinements results resulted in non-physical model and were thus excluded. The heterogeneity of the OC-pre sample is the most likely explanation for this unstable refinement, and we encourage future study into determining the structure of the Ni-rich surface phase and the Mn-rich bulk phase seen in the OC-pre sample.

Due to the inability of the stable refinement of the OC-pre sample the refinement of the XRD patterns predominately confirms the structure of the materials as R-3m and demonstrates that the materials experienced significant structural rearrangement as a result of electrochemical cycling.

Refinement was conducted using the GSAS-II program [14,15]. Refinement was conducted as follows:

1) A CIF file from crystal maker was imported and refinement was conducted on the XRD data to determine background.
2) Fine displacements were refined, with no displacement factors exceeding 400 µm.
3) Lattice parameters were then refined.
4) Particle size was then refined.
5) The atomic fractions were then refined under the following constraints:
    a) Total Lithium = 1.1667
    b) Total nickel = 0.25
    c) Total occupancy of site 1 = 1
    d) Total occupancy site 2 = 1
6) Refinement was then concluded.

SI20 – Resistance per cycle and impedance corrected average voltage per cycle of the samples

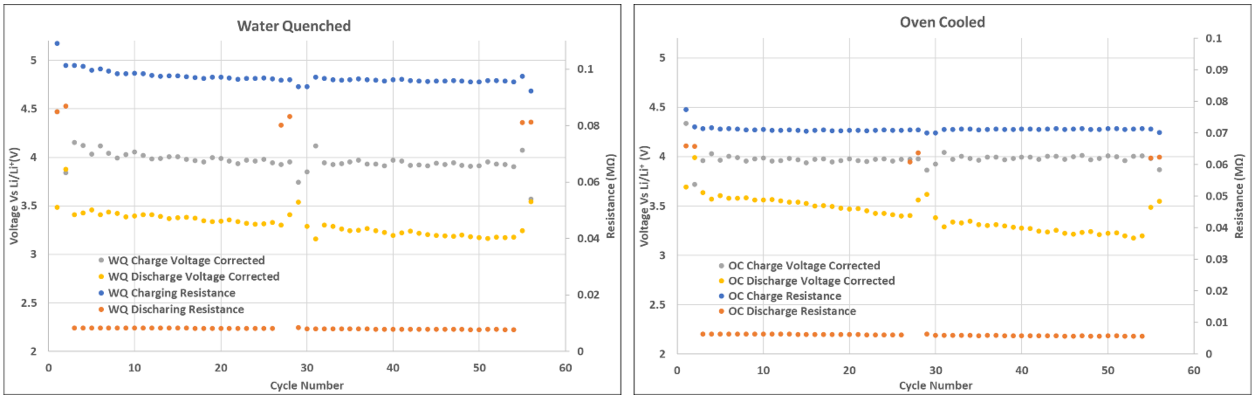

These data demonstrate that the discharge resistance of both cells was relatively similar, while the charge resistance is higher and decays faster on the WQ sample. Critically, when voltage was corrected for any impedance changes the overall voltage trends did not change.

SI21 – Demonstrating anti-site defect impact on XRD pattern's peak intensities

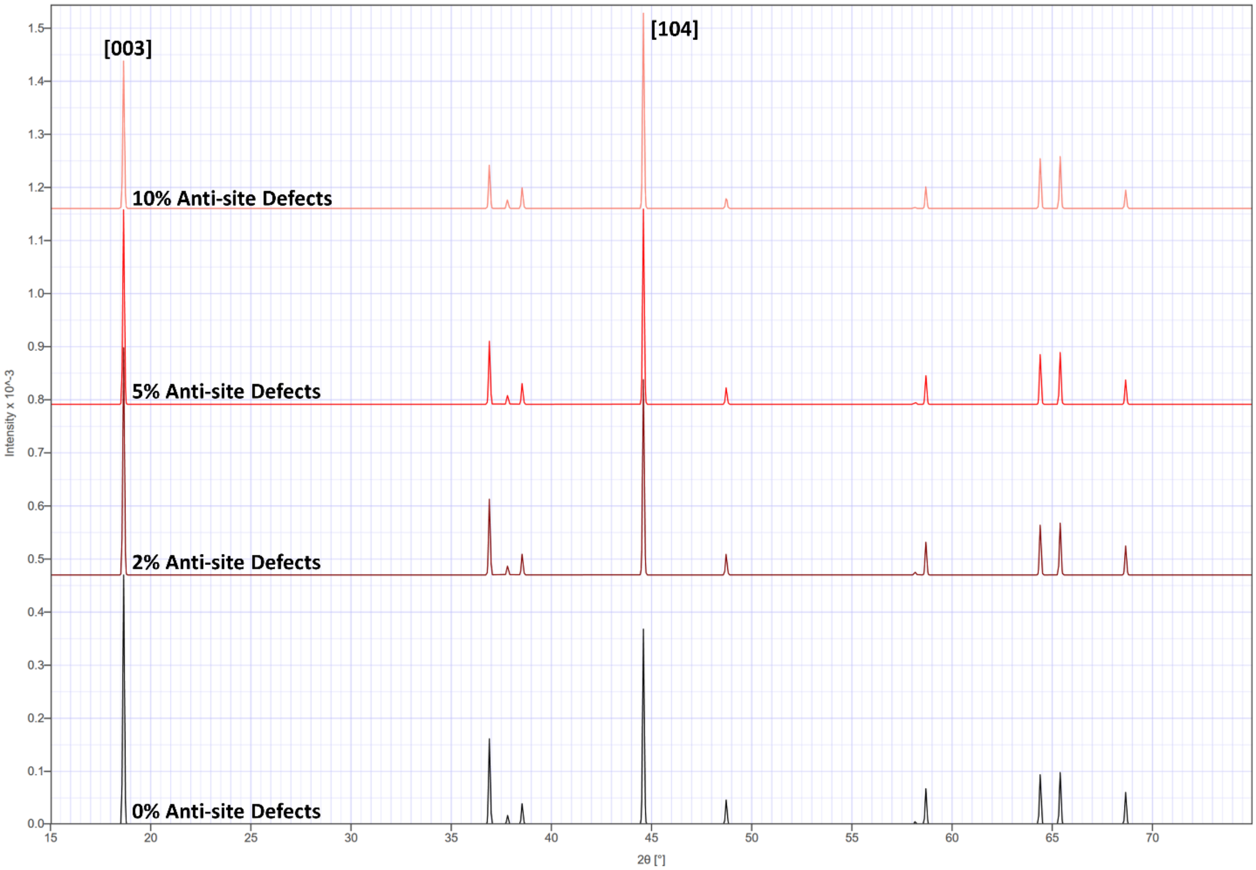

% Anti-site Defects	Relative Peak Intensity		$I_{[003]}/I_{[104]}$ Ratio
	[003]	[104]	
10%	0.757	1.000	0.757
5%	0.999	1.000	0.999
2%	1.000	0.867	1.153
0%	1.000	0.785	1.274

The figure and table shown here demonstrate how by increasing the amount of Ni^{2+} in the lithium layers of the $Li[Ni_{0.25}Li_{0.167}Mn_{0.583}]O_2$ material the relative intensity of the 104 peak increases while the relative

intensity of the 003 peak decreases. These XRD patterns were simulated in crystal diffract and were based off the model R-3m structure of the material. Note this model system did not have the superlattice order simulated, and because of this the superlattice peaks commonly seen around 20° 2θ are not present.

To demonstrate the impact of the anti-stie defects on the material, the 10% anti-site defect material's composition in the program is listed as $Li_{0.9}Ni_{0.1}[Ni_{0.15}Li_{0.267}Mn_{0.583}]O_2$, with the compositions of the others matching their listed percentage of anti-site defects.

SI bibliography:

1. Zheng, H. *et al.* Recent developments and challenges of Li-rich Mn-based cathode materials for high-energy lithium-ion batteries. *Materials Today Energy* vol. 18 Preprint at https://doi.org/10.1016/j.mtener.2020.100518 (2020).

2. Meng, Y. S. *et al.* Cation Ordering in Layered O3 Li[Ni x Li 1/3-2x/3 Mn 2/3-x/3]O 2 (0 < x <1 / 2) Compounds Three layered compounds. *Chemistry of Materials* **17**, 2386–2394 (2005).

3. Bréger, J. *et al.* Short-and Long-Range Order in the Positive Electrode Material, Li(NiMn) 0.5 O 2 : A Joint X-ray and Neutron Diffraction, Pair Distribution Function Analysis and NMR Study. *J Am Chem Soc* **127**, 7529–7537 (2005).

4. Yuan, S. *et al.* Improving the Electrochemical Performance of a Lithium-Rich Layered Cathode with an in Situ Transformed Layered@Spinel@Spinel Heterostructure. *ACS Appl Energy Mater* **4**, 11014–11025 (2021).

5. Li, S. *et al.* Enhanced Activity and Reversibility of Anionic Redox by Tuning Lithium Vacancies in Li-Rich Cathode Materials. *Cite This: ACS Appl. Mater. Interfaces* **13**, 39490 (2021).

6. Zhu, C., Hu, Y., Jiang, N., Pan, D. & Li, C. Promoting reversible reaction of oxygen anions in cobalt-free lithium-rich layered oxides to improve their electrochemical performance. *Appl Surf Sci* 150587 (2021) doi:10.1016/J.APSUSC.2021.150587.

7. Naylor, A. J. *et al.* Depth-dependent oxygen redox activity in lithium-rich layered oxide cathodes †. (2019) doi:10.1039/c9ta09019c.

8. Moulder, J. F., F., S. W., Sobol, P. E. & Bomben, K. D. *Handbook of X-ray Photoelectron Spectroscopy Edited by*. (Perkin-Elmer).

9. Aoki, A. X-ray photoelectron spectroscopic studies on ZnS: MnF2 phosphors. *Jpn J Appl Phys* **15**, 305–311 (1976).

10. National Institute of Standards and Technology. NIST X-ray Photoelectron Spectroscopy (XPS) Database. *NIST Standard Reference Database Number 20* https://srdata.nist.gov/xps/citation.aspx doi:10.18434/T4T88K.

11. Töpfer, J. *et al.* Cation Valencies and Distribution in the Spinels NiMn2O4 and MzNiMn2−zO4 (M = Li, Cu) Studied by XPS. *physica status solidi (a)* **134**, 405–415 (1992).

12. Bruce, P. G., Armstrong, A. R. & Gitzendanner, R. L. New intercalation compounds for lithium batteries: layered LiMnO 2 †. *J Mater Chem* **9**, 193–198 (1999).

13. Verhoeven, V. W. J., Mulder, F. M. & de Schepper, I. M. Influence of Mn by Li substitution on the Jahn–Teller distortion in LiMn2O4. *Physica B Condens Matter* **276–278**, 950–951 (2000).

14. Toby, B. H. & Von Dreele, R. B. *GSAS-II* : the genesis of a modern open-source all purpose crystallography software package. *J Appl Crystallogr* **46**, 544–549 (2013).

15. Rietveld, H. M. *A Profile Refinement Method for Nuclear and Magnetic Structures*. *J. Appl. Cryst* vol. 2 (1969).
